# Atomically Precise Cu Nanoclusters: Recent Advances, Challenges, and Perspectives in Synthesis and Catalytic Applications

**DOI:** 10.1007/s40820-024-01555-6

**Published:** 2024-12-03

**Authors:** Mengyao Chen, Chengyu Guo, Lubing Qin, Lei Wang, Liang Qiao, Kebin Chi, Zhenghua Tang

**Affiliations:** 1 New Energy Research Institute, School of Environment and Energy, South China University of Technology, Guangzhou Higher Education Mega Centre, Guangzhou, 510006 People’s Republic of China; 2 Petrochemical Research Institute, PetroChina Company Limited, Beijing, 102206 People’s Republic of China; 3 Key Laboratory of Functional Inorganic Material Chemistry (Heilongjiang University), Ministry of Education, Harbin, 150001 People’s Republic of China

**Keywords:** Atomically precise Cu nanoclusters, Controllable synthesis, Catalytic applications, Structure–performance relationship, Challenges and perspectives

## Abstract

Atomically precise metal nanoclusters are an emerging type of nanomaterial which has diverse interfacial metal–ligand coordination motifs that can significantly affect their physicochemical properties and functionalities. Among that, Cu nanoclusters have been gaining continuous increasing research attentions, thanks to the low cost, diversified structures, and superior catalytic performance for various reactions. In this review, we first summarize the recent progress regarding the synthetic methods of atomically precise Cu nanoclusters and the coordination modes between Cu and several typical ligands and then discuss the catalytic applications of these Cu nanoclusters with some explicit examples to explain the atomical-level structure–performance relationship. Finally, the current challenges and future research perspectives with some critical thoughts are elaborated. We hope this review can not only provide a whole picture of the current advances regarding the synthesis and catalytic applications of atomically precise Cu nanoclusters, but also points out some future research visions in this rapidly booming field.

## Highlights

- Summarizing recent advances on synthesis and catalytic applications of Cu nanoclusters.
- The structure–property–functionality relationship is clearly elucidated.
- Critical analysis of the current challenges and future perspectives.

## Graphical abstract

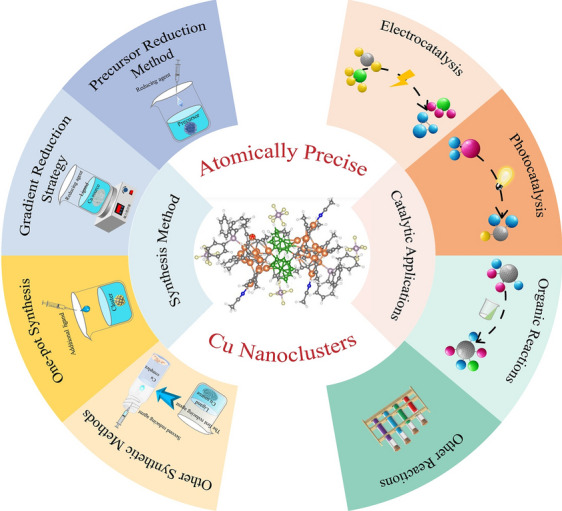

## Introduction

### Uniqueness of Atomically Precise Cu Nanoclusters

The last decade has witnessed the great success of nanoscience and nanotechnology, yet ideal research model with well-defined composition and structure is still lacking. Specifically, most of the studied nanomaterials are quite polydisperse, that means, in most studied systems, the nano-scientists are extremely difficult to find two same nanoparticles with identical size, morphology, composition, and structure. To advance nanoscience and nanotechnology, one of the ultimate goals for the nano-scientists is to find a truly uniform system, in another word, atomically precise nanoparticles as research models [1, 2].

The emergence of atomically precise coinage metal (Au, Ag, Cu, and its alloy, etc.) nanoclusters can realize such goal [1]. Atomically precise metal nanocluster is a novel type of nanomaterial with the size in the sub-nanometer regime, normally 1–3 nm in diameter. It usually comprises 10–300 metal atoms with surface ligand as the protecting agents capping on the metal core. Note that as the nanoparticle size decreases downward to the sub-nanometer range, due to the strong quantum confinement effect, distinctly different physicochemical properties of metal nanoclusters from relatively large metal nanoparticles are observed [3]. For instance, discrete optical absorbance features can be readily identified in molecular Au nanoclusters, but such feature is absent in larger Au nanoparticle counterparts [4]. More importantly, the sub-nanometer size of the metal nanocluster is still within the resolving limitation of single crystal X-ray diffraction (SC-XRD), that allows nano-chemists to resolve their structure with atomic precision [5]. Such precise structure cannot be available for many other nanomaterials, which render metal nanoclusters unique advantages to comprehensively study the structure–property relationship in various fields, such as sensing [6, 7], assembly [8, 9], catalysis [10–12], optoelectronic [13, 14], and cancer therapy [15–17]. Furthermore, the chemical stability of these metal nanoclusters in terms of electronic structure can be explained by the “superatom” theory, where the electrons are confined in the spherical metal core of a jellium model [18]. It is believed that if the free electron number of a cluster is in good agreement of inert gas atoms (e.g., 2, 8, 18, 34, 52 electrons in the outmost orbital for He, Ne, Ar, Kr, Xe), it can be considered as a superatom having robust stability [18]. Meanwhile, the thermodynamic stability of thiolate metal nanoclusters is associated with the energy balance between the adsorption strength of the ligand shell to the metal core and the cohesive energy of the metal core [19]. In addition, for the non-magic number metal nanoclusters, the Wang group developed a superatomic orbital splitting (SOS) theory to understand the electronic configuration, where the shape of the metal core is considered in determining the order of the group orbital levels [20].

It is worth noting that, compared with noble metal nanoclusters such as Au, Ag, Pd, and Pt, Cu nanoclusters possess some unique characteristics. First, Cu is more earth abundant hence can be more cost effective for large-scale production to prepare functional nanomaterials. Secondly, Cu possesses different valence states including 0, +1, +2, which render Cu atom with different spatial arrangement in the metal core. So far, superatomic Cu nanoclusters with Cu(0) core, Cu(I) clusters, and Cu(II) complexes have been extensively reported [21, 22]. It is worth noting that different charge states impart Cu nanomaterial with drastically different physicochemical properties and reactivities, and the variation of the valence state during some catalytic reaction has also been observed [23–25]. Finally, Cu nanoclusters hold some peculiar functionalities that are not available from other noble metal nanoclusters [26]. For instance, Cu has good adsorption for CO_2_ than hydrogen, and it has strong capability to construct C–C bond, so in electrochemical CO_2_ reduction, using Cu nanoclusters can suppress the hydrogen evolution reaction and obtain HCO_2_H, CH_3_OH, CH, and even more valuable C2+ products such as C_2_H_4_ and C_2_H_5_OH [27], while only CO can be acquired when using Au or Ag nanoclusters as catalysts in most cases [28–34]. Table 1 summarizes all the Cu nanoclusters which have been discussed in this paper.

**Table 1:** The formula, metal core configuration, and catalytic reaction of Cu nanoclusters discussed in this paper

Formulas	Cluster configuration	Catalytic reaction	Refs.
[Cu_6_(MBD)_6_]	Octahedron	CO_2_RR	[27]
[Cu_29_Cl_4_H_22_(Ph_2_phen)_12_]Cl	Cu_13_@Cu_16_	–	[35]
[Cu_20_(C**≡**CPh)_12_(OAc)_6_)]	Cu_4_@Cu_16_	[3 + 2] Cycloaddition	[36]
[Cu_53_(C**≡**CPhPh)_9_(dppp)_6_Cl_3_(NO_3_)_9_]	Layer-structure	–	[37]
[Cu_26_(DPPE)_3_(CF_3_CO_2_)_8_(CH_3_O)_2_(^t^BuC**≡**C)_4_H_11_]^+^	Cu_4_@Cu_6_@Cu_16_	CO_2_RR	[38]
[Cu_32_(PET)_24_H_8_Cl_2_](PPh_4_)_2_	Cu_18_@Cu_4_PET_2_@ Cu_14_(PET)_22_Cl_2_	Carbonylation of aniline	[39]
[Cu_25_H_22_(PPh_3_)_12_]Cl	Cu_13_@Cu_12_	–	[40]
[Cu_18_H_17_(PPh_3_)_10_]Cl	Cu_8_@Cu_10_	–	[41]
[Cu_18_H_3_(S-Adm)_12_(PPh_3_)_4_Cl_2_]	Cu_10_@Cu_8_S_12_P_4_	–	[42]
[Cu_31_(4-MeO-PhC**≡**C)_21_(dppe)_3_](ClO_4_)_2_	Cu_13_@Cu_18_	–	[43]
[Cu_6_(4-MeO-PhC**≡**C)_5_(dppe)_3_](ClO_4_)	Trigonal-prismatic configuration	–	[44]
[Cu_50_(CF_3_COO)_12_(3, 5-*di*Me-PhS)_18_(PPh_3_)_4_H_2_]	Cu_44_@Cu_6_	–	[45]
[Cu_41_Cl_2_(2-F-C_6_H_4_S)_12_(CF_3_COO)_6_(PPh_3_)_6_H_19_]^2−^	Cu_29_@Cu_12_	*p*-Nitrophenol reduction	[46]
[Cu_41_(2, 5-di-Methyl-C_6_H_3_S)_12_(BO_3_)_3_Cl_3_(PPh_3_)_6_H_19_]	Cu_29_@Cu_12_	*p*-Nitrophenol reduction	[46]
[Cu_23_(^t^BuC**≡**C)_13_(CF_3_COO)_6_]	Cu_4_@Cu_19_	–	[47]
[Cu_23_(^t^BuC**≡**C)_13_(CF_3_COO)_6_]**·**CHCl_3_	Cu_4_@Cu_19_	–	[47]
[Cu_53_(RCOO)_10_(C**≡**C^t^Bu)_20_Cl_2_H_18_]^+^	Cu_3_@Cu_10_Cl_2_@Cu_20_@Cu_20_	–	[48]
[Cu_13_Na_2_(CZ-PrA)_6_(TC4A)_2_Cl(CH_3_OH)_2_]	Cu_9_@Cu_4_	Sulfide oxidation	[49]
[Cu_13_Na(CZ-PrA)_6_(TC4A)_2_(CH_3_OH)]·CH_3_OH·CH_2_Cl_2_·CH_3_COCH_3_	Cu_9_@Cu_4_Cl	Sulfide oxidation	[49]
[Cu_13_(S_2_CN^n^Bu_2_)_6_(C**≡**CR)_4_](PF_6_)	Cu@Cu_12_ cuboctahedron	–	[50]
[Cu_32_H_20_(S_2_P(O^i^Pr)_2_)_12_]	Cu_14_@Cu_9_@Cu_9_	CO_2_RR	[51]
[Cu_8_(H)(L1)_6_PF_6_] (L1 = 9H-carbazole-9-carbodithioate)	Cu_4_ double tetrahedron	CO_2_RR	[52]
[Cu_8_(^t^BuS)_4_(L1)_4_] (L1 = 9H-carbazole-9-carbodithioate	Cu_4_ double tetrahedron	CO_2_RR	[52]
[Cu_8_(^*t*^BuS)_4_(L2)_4_] (L2 = *O*-ethyl carbonodithiolate)	Cu_4_ double tetrahedron	CO_2_RR	[52]
[Cu_13_(C_4_B_10_H_11_)_10_(PPh_3_)_2_(CH_3_CN)_2_](PF_6_)_3_	–	Nitrate reduction	[53]
[Cu_26_(C_4_B_10_H_11_)_16_(C_2_B_9_H_10_C_2_)_2_(PPh_3_)_2_(CH_3_CN)_4_](PF_6_)_4_	–	Nitrate reduction	[53]
[Cu_6_(HL_1_)_2_(L1)_4_(PF_6_)_2_]	Cu_6_ octahedron	CO_2_RR	[54]
[Cu_61_(S^t^Bu)_26_S_6_Cl_6_H_14_]	Cu_19_@Cu_42_(S^t^Bu)_26_S_6_Cl_6_	–	[55]
[Cu_57_H_20_(PET)_36_(TPP)_4_]^+^	Cu_14_@Cu_43_S_36_	–	[56]
[Cu_58_H_20_PET_36_(PPh_3_)_4_]^2+^	Cu_8_@Cu_6_@Cu_24_@Cu_12_ @Cu_8_	[3 + 2] Cycloaddition	[57]
[Cu_57_H_20_PET_36_(PPh_3_)_4_]^+^	Cu_8_@Cu_6_@Cu_24_@Cu_12_ @Cu_7_	[3 + 2] Cycloaddition	[57]
[Cu_28_H_10_(C_7_H_7_S)_18_(TPP)_3_]	Cu_13_@Cu_15_S_18_P_3_	C−C coupling reaction	[58]
[Cu_7_(SC_5_H_9_)_7_(PPh_3_)_3_]	–	C−C coupling reaction	[59]
[Cu_3_(NHC)_3_(PF_6_)_3_]	–	A^3^ coupling and Redox-A^3^ coupling reaction	[60]
[Cu_8_(Tf-dpf)_4_(NO_3_)_2_](NO_3_)_2_	Two thin slices of supramolecular layers	“Aldehyde–acetylene–amine” A^3^ coupling reaction	[61]
[Cu_8_(L2)_2_(L3)_2_](L2, L3 = deprotonated pyrazole)	–	Synthesis of indolizine	[62]
Cu_18_H(PET)_14_(PPh_3_)_6_(isothiocyanate)_3_	Cu_15_@Cu_3_	Reduction of ferricyanide to ferrocyanide	[63]
[Cu_66_Cl_8_(PPh_3_)_8_(SC_2_H_5_)_32_H_24_](SbF_6_)_2_	Layered arrangement	Hydrogenation of cyclohexanone	[64]
[Cu_20_H_9_(Tf-dpf)_10_]·BF_4_	Cu_12_@2Cu_4_	Conjugate reduction of cinnamaldehyde	[65]
[Cu_20_H_8_(Tf-dpf)_10_]·(BF_4_)_2_	Cu_12_@2Cu_4_	Conjugate reduction of cinnamaldehyde	[65]
Se@Cu_20_(PhSe)_12_(PPh_3_)_2_(C_6_H_5_COO)_6_	Se@Cu_6_@Cu_3_@Cu_3_@Cu_3_@Cu_3_@Cu_2_	4-Nitrophenol reduction	[66]
Se@Cu_20_(PhSe)_12_(PPh_3_)_2_(CF_3_COO)_6_	Se@Cu_6_@Cu_3_@Cu_3_@Cu_3_@Cu_3_@Cu_2_	4-Nitrophenol reduction	[66]

– denotes none or not available

### Cu–Ligand Coordination Modes in Cu Nanoclusters

**Scheme 1:**
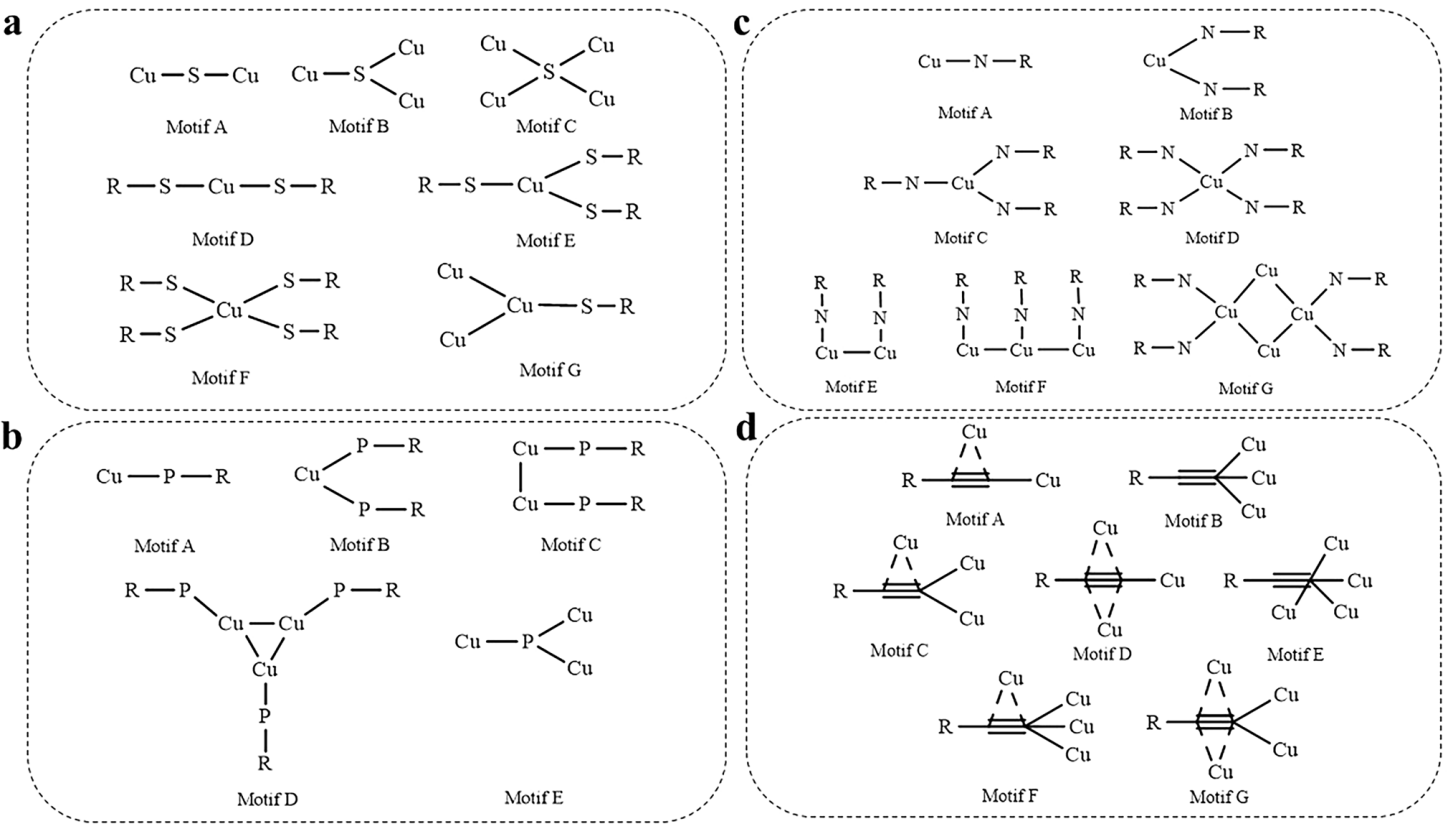
Metal–ligand coordination modes in Cu nanoclusters. **a** Cu–S coordination modes, **b** Cu–P coordination mode, **c** Cu–N coordination mode, **d** Cu–alkynyl carbon coordination mode

To prevent the Cu atoms from agglomeration or aggregation, surface capping ligand is critical as the stabilizing agent to allow Cu atoms to form a certain small core but not into a bulky core or an agglomerate. Furthermore, the ligand can significantly affect the physical and chemical properties of Cu nanoclusters through binding with the surface Cu atoms [67–69]. These interfacial coordination moieties form the ligament between the metal core and the surface ligands, and such ligament governs the electron coupling behaviors. So far, various types of molecules have been employed as capping ligand to stabilize the Cu core, and four typical widely employed organic molecules are thiolate [1, 3], phosphine containing molecule [70], nitrogen containing molecule [71], and alkynyl ligand [72, 73], yet they have markedly different coordination modes to bind the Cu atoms (Scheme 1). As illustrated in Scheme 1a, one S atom can coordinate with one, two, three, and four Cu atoms [39], and one Cu atom can coordinate with one, two, three, and four S atoms [74, 75]. One Cu atom can coordinate with one or two P atoms [40, 41], and one P atom can coordinate with three Cu atoms (Scheme 1b). Interestingly, three Cu atoms and three P atoms can form a cyclic triangle coordination mode [76]. The coordination mode between N and Cu is somewhat similar to that between P and Cu, as one Cu atom can coordinate with one or two P atoms, and both N and P atoms can form the dimer mode of R–N(P)–Cu (Scheme 1c) [21, 35, 77, 78]. However, unlike P, one N atom cannot coordinate with two or more Cu atoms, but it can form the trimer mode of R–N–Cu, and a cyclic quadrangle mode of Cu_4_N_4_ is also available [77]. It is worth noting that, for S, N, P atoms, they coordinate with the Cu atom mainly through σ bonding, nevertheless, for alkynyl ligand, it can coordinate with Cu atom with either σ bonding or π bonding or both. As shown in Scheme 1d, one alkynyl ligand can not only coordinate with three or four Cu atoms with only σ bonding, but also can coordinate with two, three, or four Cu atoms with both σ bonding and π bonding [36, 37, 79, 80]. There are seven essential types of coordination modes: *μ*_2_-*ƞ*^1^, *ƞ*^2^; *μ*_3_-*ƞ*^1^, *ƞ*^1^, *ƞ*^1^; *μ*_3_-*ƞ*^1^, *ƞ*^1^, *ƞ*^2^; *μ*_3_-*ƞ*^1^, *ƞ*^2^, *ƞ*^2^; *μ*_4_-*ƞ*^1^, *ƞ*^1^, *ƞ*^1^, *ƞ*^1^; *μ*_4_-*ƞ*^1^, *ƞ*^1^, *ƞ*^1^, *ƞ*^2^; *μ*_4_-*ƞ*^1^, *ƞ*^1^, *ƞ*^2^, *ƞ*^2^. Such unique binding modes can impart alkynyl-protected Cu nanoclusters with some drastically different physicochemical properties and functionalities from thiolate, nitrogen, and phosphine ligand stabilized Cu nanoclusters [72, 73].

## Synthesis of Atomically Precise Cu Nanoclusters

There are various methods to synthesize bulky metal nanoparticles. However, preparing metal nanoclusters with atomic precision is still quite challenging. This is because metal nanoclusters with molecular purity typically form under specific thermodynamic or kinetic conditions. Since the pioneering Brust method to prepare thiolate-protected Au nanoclusters reported at 1990s [81, 82], a number of methods have been developed to prepare Au nanoclusters or Ag nanoclusters protected by thiolate, alkynyl molecule, and other ligands. The typical approaches include, but are not limited to, the direct reduction of the precursor, one-pot strategy, bi-phase method, ligand exchange or etching and so on [4, 83–87]. It is worth noting that, to prepare monodisperse metal nanoclusters, there are a lot of factors influencing the output, e.g., the reaction temperature, the solvent, the reducing agent, the nature and chemical property of the ligand molecule, the stoichiometric ratio of ligand–reductant–reactant, and so on. For instance, our group developed a synchronous nucleation and passivation strategy to synthesize alkynyl-protected coinage metal nanoclusters [88], and so far, several Au [88, 89], Ag [29, 32], AuAg [90–92], AgPd [93], AgRh [94, 95] nanoclusters have been successfully fabricated by this method [73, 96], but this approach is not applicable to thiolate-protected metal nanoclusters. Meanwhile, no successful case has been achieved on Cu nanoclusters yet. It also implies that, the approaches developed for synthesizing molecular thiolate-protected metal nanoclusters or alkynyl-protected metal (and alloy but not pure Cu) nanoclusters are not applicable for preparing atomically precise Cu nanoclusters, probably due to the different metal–ligand interactions can alter the nuclei growth and surface passivation behaviors [97].

**Fig. 1:**
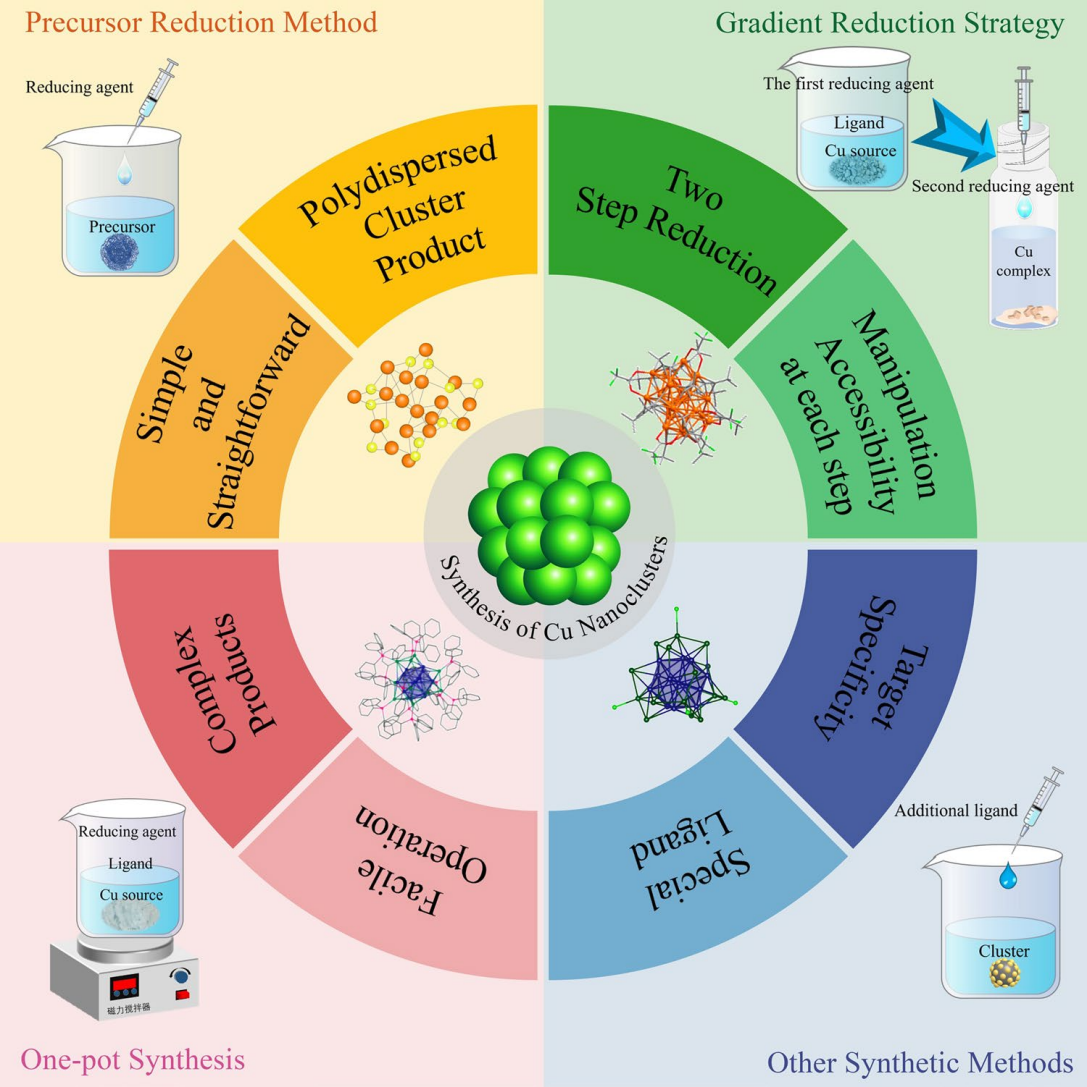
Current synthetic methods for atomically precise Cu nanoclusters

Nevertheless, several generic methods have been developed for synthesizing monodisperses or molecular Cu nanoclusters. It includes the precursor reduction method, gradient reduction strategy (GRS), one-pot synthesis (OPS), ligand-exchange-induced growth, and other ingenious methods (Fig. 1). Each method has its own advantages or disadvantages. For example, the precursor reduction method is quite straightforward, but polydisperse cluster product may be acquired; the gradient reduction strategy features two step reduction with manipulation accessibility at each step, but the overall yield might be quite low; the one-pot synthesis holds facile operation but complex product is highly possible; the other methods are more applicable to special ligand to target specific cluster molecule. The advantages and disadvantages of these methods with specific cases will be elaborated next.

### Precursor Reduction Method

**Scheme 2:**
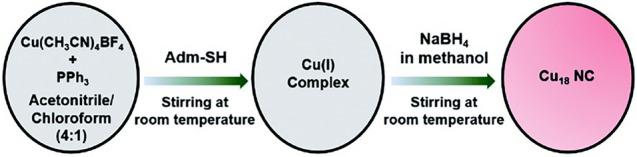
Synthetic route of Cu_18_ nanoclusters. Reproduced with permission from Ref. [42], Copyright 2022 Royal Society of Chemistry

The simplest and most straightforward method to fabricate Cu nanoclusters is to form the precursor first and then reduce it. However, under most circumstances, polydispersed Cu nanoclusters with a wide size distribution are acquired. To improve the monodispersity, kinetic control is inevitable. One typical example is the synthesis of [Cu_18_H_3_(S-Adm)_12_(PPh_3_)_4_Cl_2_] molecule reported by Mandal group [42]. As shown in Scheme 2, Cu(CH_3_CN)_4_BF_4_ was treated with auxiliary PPh_3_ ligand first, and then the adamantanethiol (Adm-SH) ligand was added to form the Cu(I) complex. Upon the addition of the NaBH_4_ methanol solution, the mixture changed from colorless into red, indicating the Cu_18_ nanocluster was formed. Note that, the employment of the bulky Adm-SH ligand is critical for yielding the Cu_18_ nanocluster here, as it acts as the main surface protecting ligand to stabilize the framework of the Cu_18_ nanocluster [42]. The Cu(I) precursor, also called as the Cu(I) complex, can be prepared step by step. In 2020, Li et al. reported a copper hydride cluster of [Cu_32_(PET)_24_H_8_Cl_2_](PPh_4_)_2_ (PET = phenylethyl thiolate) [39]. For its synthesis, Cu(TMEDA)Cl (TMEDA: tetramethylethylenediamine) was first obtained by reacting CuCl with TMEDA. Upon addition of PPh_4_Br and the thiol ligand (PETH), the final stage precursor of CuPET(TMEDA)Cl was obtained. The reduction of CuPET(TMEDA)Cl by sodium borohydride can yield Cu_32_ nanoclusters [39]. In 2023, Jia et al. utilized this method to fabricate an eight-electron superatom of [Cu_31_(4-MeO-PhC**≡**C)_21_(dppe)_3_](ClO_4_)_2_ (Cu_31_, dppe = 1, 2-bis(diphenylphosphino)ethane) cluster [43]. Specifically, Cu(ClO_4_)_2_**·**6H_2_O reacts with 4-methoxyphenylacetylene and dppe in the presence of triethylamine to form the precursor first, then Cu_31_ nanoclusters were obtained upon the reduction of borohydride [43]. Interestingly, side product of [Cu_6_(4-MeO-PhC**≡**C)_5_(dppe)_3_](ClO_4_) (it is a Cu(I) cluster not a superatom) was also isolated from the obtained single crystals of the Cu_31_ nanoclusters [44]. Recently, Fang, Wei and Shen et al. documented the fabrication of four Cu_50_ clusters with nearly identical metal frameworks [45]. Using Cu_50_(CF_3_COO)_12_(3, 5-*di*Me-PhS)_18_(PPh_3_)_4_H_2_ (Cu_50_-1) as an example, Cu(CF_3_COO)_2_ was first prepared as the Cu source, and after it reacted with 3, 5-dimethylbenzenethiol and PPh_3_, NaBH_4_ was added as a reducing agent to obtain the raw cluster product [45].

It is worth noting that, after forming the raw product by the precursor reduction method, adding some auxiliary ligand to etch the clusters may help to improve the yield and stability of the final product. For instance, Shen and coworkers recently reported a couple of “isostructural” Cu clusters of [Cu_41_Cl_2_(2-F-C_6_H_4_S)_12_(CF_3_COO)_6_(PPh_3_)_6_H_19_]^2−^ and [Cu_41_(2, 5-di-Methyl-C_6_H_3_S)_12_(BO_3_)_3_Cl_3_(PPh_3_)_6_H_19_] [46]. The raw product was formed by reacting Cu(CF_3_COO)_2_ with 2-fluorothiophenol and PPh_3_ ligand, but after that, additional PPh_3_Cl ligand was added to introduce Cl onto the two Cu_41_ clusters for enhanced stability [46].

### Gradient Reduction Strategy (GRS)

The gradient reduction strategy was first employed by the Zheng group to synthesize Cu_53_ nanoclusters, but the method was not named then in 2019 [48]. Specifically, Cu(CF_3_COO)_2_ was first reduced by Cu powder, after the tert-butylacetylene (HC**≡**C^t^Bu) ligand was added, PhSiH_2_ as the second reducing agent was introduced into the solution under vigorous stirring. It is believed that the cooperation between Cu powder and PhSiH_2_ contributed together to achieve the high-nuclearity Cu(I)/Cu(0) cluster of [Cu_53_(RCOO)_10_(C**≡**C^t^Bu)_20_Cl_2_H_18_]^+^ [48]. In another study, the synthesis and overall structure of Cu_53_(C**≡**CPhPh)_9_(dppp)_6_Cl_3_(NO_3_)_9_ nanocluster was recorded by Li and Zhang group. In a typical trial, 4-ethynylbiphenyl (BP) and 1, 3-bis(diphenylphosphino)propane (dppp) were sequentially added into an ethanol solution of Cu(NO_3_)_2_, then NaBH_4_ was introduced, followed by aging the reaction to obtain the target cluster [37].

**Scheme 3:**
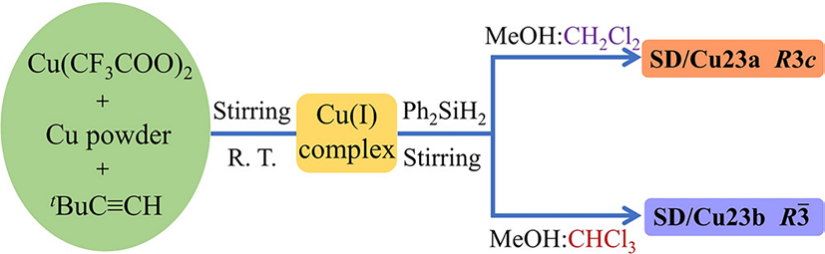
Synthetic route of Cu23a and Cu23b nanoclusters by GRS. Reproduced with permission from Ref. [47], Copyright 2020 American Chemical Society

In 2020, the name of gradient reduction strategy was formally proposed by Sun group [47]. In this method, the valence of the Cu atom evolved from +2 to +1, then to 0 by using different reducing agents at different stages. As illustrated in Scheme 3, the comproportionation of Cu(II) and Cu powder can yield the Cu(I) complex first, then the Cu(I) complex was further reduced to generate [Cu_23_(^t^BuC**≡**C)_13_(CF_3_COO)_6_] (SD/Cu23a) or [Cu_23_(^t^BuC**≡**C)_13_(CF_3_COO)_6_]**·**CHCl_3_ (SD/Cu23b). Such method showed the solvent-dependent polymorphism. It is worth noting that the Cu_23_ nanoclusters are superatoms, not Cu(I) complex, and both of them have four valent electrons. They also contain a very rare [Cu_4_]^0^ kernel surrounded by an outer Cu_19_ shell. In addition, depending on the solvent, the Cu_23_ nanocluster can crystallize into two polymorphs [47]. In 2022, the same group conducted the synthesis of two quasi-structurally isomeric 13-nuclei Cu nanoclusters (Cu13a and Cu13b) by the GRS method in a similar manner [49]. In this study, after the reaction of Cu(II) and CZ-PrAH (9-(prop-2-yn-1-yl)-9H-carbazole) with Cu powder to yield a yellow Cu(I) intermediate, the ligand was added into the mixture, following that a freshly prepared ethanol solution of NaBH_4_ was added under vigorous stirring. The reducing agent of NaBH_4_ with strong reducing capability was employed to avoid the re-oxidation of Cu(I) species [49]. Recently, Li et al. reported the comprehensive characterization and electrocatalytic CO_2_ reduction of [Cu_26_(DPPE)_3_(CF_3_CO_2_)_8_(CH_3_O)_2_(^t^BuC**≡**C)_4_H_11_]^+^, which was also synthesized by this GRS approach [38].

### One-Pot Synthesis (OPS)

Compared to the above two methods, one-pot synthesis is the most facile method to operate, as it does not require to form the Cu(I) precursor. Basically, all the reactants including the Cu salt, the ligand molecules, the reducing agent and the base are mixed to generate the Cu nanoclusters. Of course, the Cu(I) precursor is formed in the reaction system, but no isolation is necessary. It is known that such method has been employed for synthesizing quite a number of coinage metal nanoclusters, e.g., thiolate Au_25_ nanoclusters can be easily prepared by one-pot approach [98, 99], the Tsukuda group fabricated a series of alkynyl-protected Au_22_ clusters by this method [100], and the Wang group reported the preparation of Au_23_(C**≡**CR)_15_ through one-pot synthesis as well [101].

**Scheme 4:**
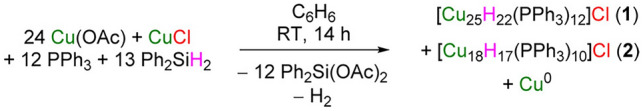
Synthetic route of Cu_25_ and Cu_28_ nanoclusters. Reproduced with permission from Ref. [40], Copyright 2015 American Chemical Society

It is worth noting that making Cu superatom cluster is rather different from making Au and Ag superatoms, as Cu(I) is more resistant to reduction. Therefore, in the presence of hydride source, a reduction can easily yield Au or Ag superatoms [2, 102], but Cu(I)-hydride complex is usually formed. It is more difficult to observe Cu(0) clusters, mainly due to the higher stability of Cu(I)-hydride complex than Au(I)-hydride or Ag(I)-hydride complexes. However, in a ligand-deficient environment, the reduction of Cu(I)-hydride complex might generate some unstable (CuH)_x_ species that are amenable for cluster growth. In 2015, the Scott and Hayton group recorded the synthesis of [Cu_25_H_22_(PPh_3_)_12_]Cl and [Cu_18_H_17_(PPh_3_)_10_]Cl by this method [40]. As summarized in Scheme 4, adding 13 equiv. of Ph_2_SiH_2_ to a slurry containing 24 equiv. of Cu(OAc), 12 equiv. of PPh_3_, and 1 equiv. of CuCl in C_6_H_6_ can result in a rapid color change from pale green to dark red then into deep green, concomitant with the precipitation of a dark brown solid after keeping stirring in 24 h. Two clusters of [Cu_25_H_22_(PPh_3_)_12_]Cl and [Cu_18_H_17_(PPh_3_)_10_]Cl were isolated with a yield of 23% and 14%, respectively [40]. Following this study, the same group accomplished the synthesis of [Cu_20_(C**≡**CPh)_12_(OAc)_6_)] by the same approach [36].

### Other Synthetic Methods

**Scheme 5:**
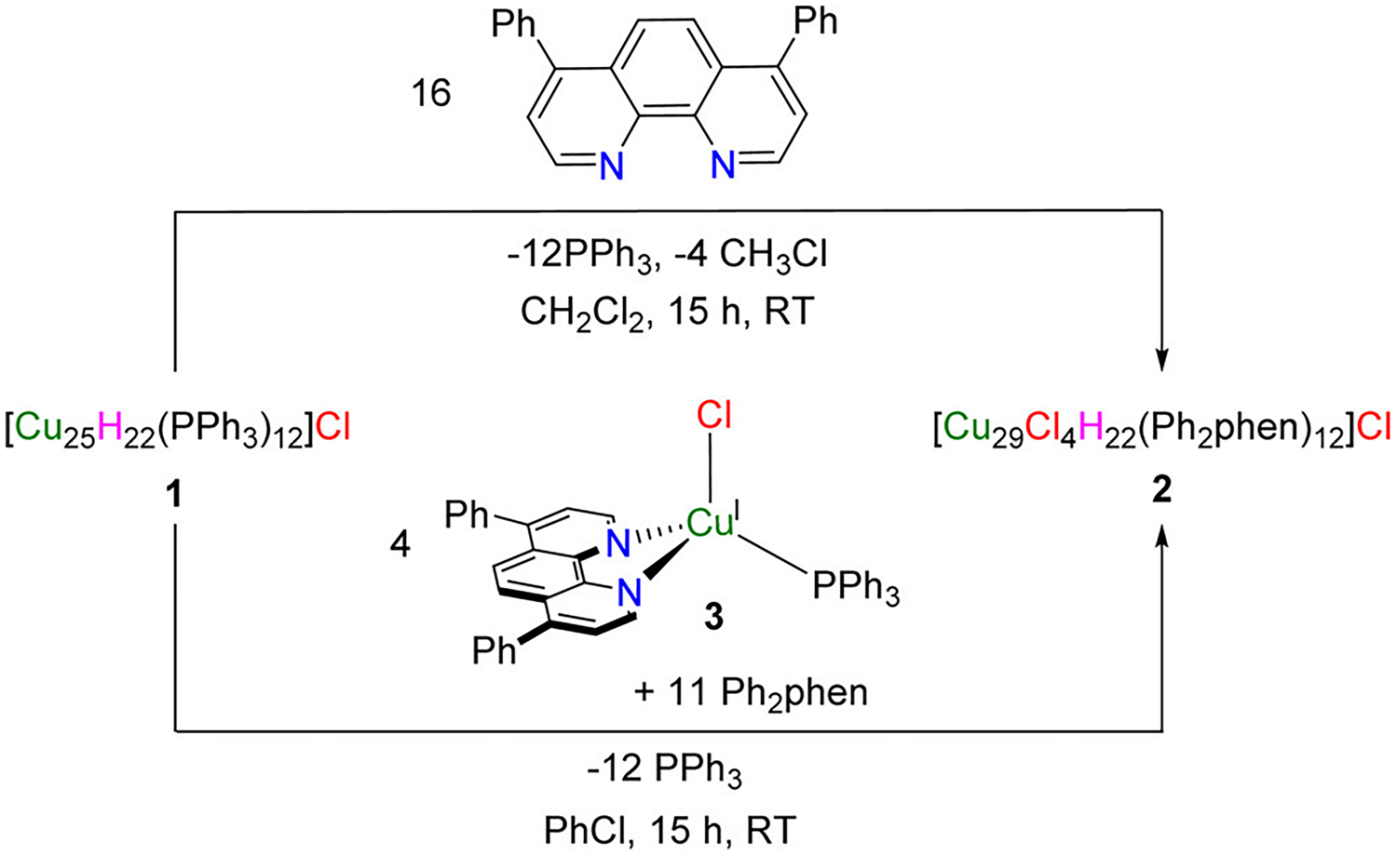
Two cluster expansion synthetic routes for Cu_29_ nanoclusters. Reproduced with permission from Ref. [35], Copyright 2016 American Chemical Society

Besides the above approaches, researchers have developed several other ingenious methods to prepare atomically precise Cu nanoclusters. In 2016, the Hayton group reported a ligand-exchange-induced growth from a smaller cluster to synthesize Cu_29_ nanoclusters [35]. As shown in Scheme 5, [Cu_25_H_22_(PPh_3_)_12_]Cl was first synthesized, and then the addition of 16 equiv of 4, 7-diphenyl-1, 10-phenanthroline (Ph_2_phen) can cause an immediate color change from dark green to dark blue, and workup of this mixture for 15 min resulted in the isolation of [Cu_29_Cl_4_H_22_(Ph_2_phen)_12_]Cl, a deep blue black crystalline material in a yield of 84%. Meanwhile, the reaction of [Cu_25_H_22_(PPh_3_)_12_]Cl with 1, 10-phenanthroline also yielded a deep blue molecule, which is probably the isostructural molecule of [Cu_29_Cl_4_H_22_(Ph_2_phen)_12_]Cl, but no single crystal XRD data were available to confirm that [35].

**Scheme 6:**
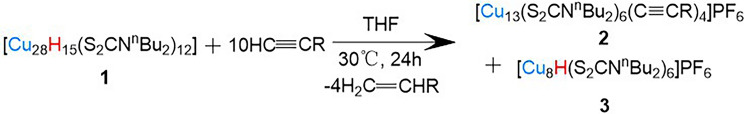
Synthesis of [Cu_13_(S_2_CN^n^Bu_2_)_6_(C**≡**CR)_4_](PF_6_) cluster. Reproduced with permission from Ref. [103], Copyright 2016 Wiley VCH

Note that the introduction of additional ligand does not necessarily lead to cluster growth or size expansion; instead, cluster size decrease is also possible. Chakrahari et al. reported the [Cu_13_(S_2_CN^n^Bu_2_)_6_(C**≡**CR)_4_](PF_6_) (R=C(O)OMe, C_6_H_4_F) cluster which was synthesized through size transformation (Scheme 6) [103]. As the hydride in [Cu_28_H_15_(S_2_CN^n^Bu_2_)_12_]^+^ is substantially hydridic, the terminal alkyne is sufficiently acidic enough to react with it to yield [Cu_13_(S_2_CN^n^Bu_2_)_6_(C**≡**CR)_4_]^+^, which features a centered cuboctahedral [Cu_13_]^11+^ core with two free electrons, and [Cu_8_H(S_2_CN^n^Bu_2_)_6_(C**≡**CR)_4_]^+^ was also isolated as the side product [103].

## Atomically Precise Cu Nanoclusters for Catalytic Applications 

**Fig. 2:**
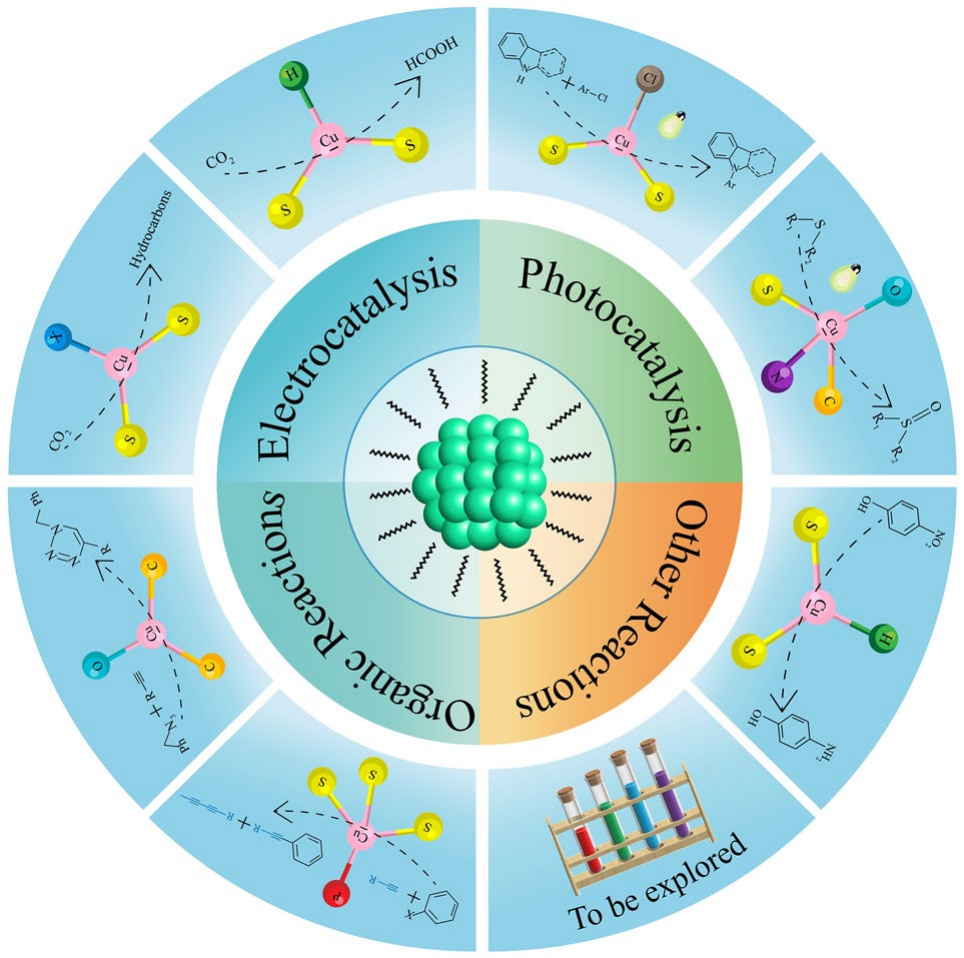
Catalytic applications of atomically precise Cu nanoclusters

Thanks to the precise composition and structure of Cu nanoclusters, they can serve as ideal model catalysts for a variety of reactions [50]. Also, theoretical simulations can build precise models to reveal the reaction pathway and help to elucidate the reaction mechanism [104, 105]. In line with this, a great deal of research effort has been devoted to exploring the catalytic applications of atomically precise Cu nanoclusters, including but not limited to electrocatalytic reactions, photocatalytic reactions, organic reactions, as well as other catalytic processes (Fig. 2).

### Cu NCs for Electrocatalysis

The electrocatalysis technology has drawn widespread attention and found immerse values in a wide spectrum of fields especially in green energy storage and conversion, as electrocatalysis is not only highly efficient, mild condition operational, multifunctional, but also can couple with renewable intermittent energy sources such as solar, wind, and geothermal energy [106]. Therefore, electrocatalysis represents a sustainable strategy to address global environmental pollution and heavy reliance on fossil fuels [107].

In the last decade, the fast industrialization and huge demand for fossil fuels have caused a dramatic worldwide increase of CO_2_ emission, which is the main culprit of global warming. However, CO_2_ is an abundant source that can be used as substrate to fabricate various valuable products such formic acid, formamide, urea, and other hydrocarbon compounds [108, 109]. Electrochemical CO_2_ reduction reaction (eCO_2_RR) can realize such conversion with high efficiency and in an environmentally friendly manner; however, due to the chemical inertness and thermodynamic stability of linear CO_2_ molecules, a prerequisite for achieving CO_2_ reduction lies in finding a catalyst that can lower the chemical energy required to break the C-O bond [110, 111]. Moreover, eCO_2_RR involves multiple electron transfer, and various products including C1 compounds (e.g., CO, HCO_2_H, CH_3_OH) and C2 compounds (e.g., CH_3_CO_2_H, CH_3_CHO, C_2_H_4_, C_2_H_6_, etc.) can be generated. The thermodynamic potentials, various reactions, and corresponding products are summarized in Table 2 [112]. It can be noted that different reactions occur at very similar thermodynamic equilibrium potentials, plus the competing hydrogen evolution reaction (HER), it is extremely challenging to achieve highly selective CO_2_ reduction product. Therefore, designing efficient, durable, and high-selectivity catalysts for eCO_2_RR to acquire target product is highly desired [113].

**Table 2:** Electrochemical CO_2_ reduction reactions yielding various products with thermodynamic equilibrium potentials in an aqueous electrolyte at pH 7, 1 atm, and 25 °C, relative to the reversible hydrogen electrode (RHE)

Reactions	*E*_0_ (V *vs*. RHE)	Product
CO_2_ + 2H^+^ + 2e^−^ → CO _(g)_ + H_2_O	− 0.11	Carbon monoxide
CO_2_ + 2H^+^ + 2e^−^ → HCOOH _(aq)_	− 0.12	Formic acid
CO_2_ + 6H^+^ + 6e^−^ → CH_3_OH _(aq)_ + H_2_O	0.03	Methanol
CO_2_ + 8H^+^ + 8e^−^ → CH_4__(g)_ + 2H_2_O	0.17	Methane
2CO_2_ + 10H^+^ + 10e^−^ → CH_3_CHO _(aq)_ + 3H_2_O	0.06	Acetaldehyde
2CO_2_ + 12H^+^ + 12e^−^ → C_2_H_4 (aq)_ + 4H_2_O	0.08	Ethylene
2CO_2_ + 12H^+^ + 12e^−^ → C_2_H_5_OH _(aq)_ + 3H_2_O	0.09	Ethanol
2CO_2_ + 14H^+^ + 14e^−^ → C_2_H_6 (aq)_ + 4H_2_O	0.14	Ethane
2CO_2_ + 16H^+^ + 16e^−^ → C_2_H_5_CHO _(aq)_ + 5H_2_O	0.09	Propionaldehyde
3CO_2_ + 18H^+^ + 18e^−^ → C_3_H_7_OH _(aq)_ + 5H_2_O	0.1	Propanol
2H^+^ + 2e^−^ → H_2_	0	Hydrogen evolution reaction
2H_2_O → O_2_ + 4H^+^ + 4e^−^	1.23	Oxygen evolution reaction

So far, all kinds of nanostructured materials including metal oxides, metal alloys, carbon substrates, two-dimensional nanomaterials have been investigated as catalysts for eCO_2_RR [114–118]. Among a series of metal-based catalysts, Cu nanomaterials are one of the most promising catalysts that can deep electrochemically reduce CO_2_ into valuable chemicals, especially hydrocarbon products [119–122]. However, for most Cu-based catalysts, the chemical nature is not uniform, e.g., no homogeneous size, shape, architecture, and structure hence it is extremely challenging to ascertain the chemical nature of the catalyst and eventually establish the structure–activity relationship [123–125]. Recently, molecular Cu nanoclusters have been drawing attention from the heterogeneous catalysis field, thanks to the high surface-to-volume ratio, strong binding capability to the key reaction intermediates, and more importantly, the atomically precise crystallographic structure and well-defined architecture [50, 90, 126].

**Fig. 3:**
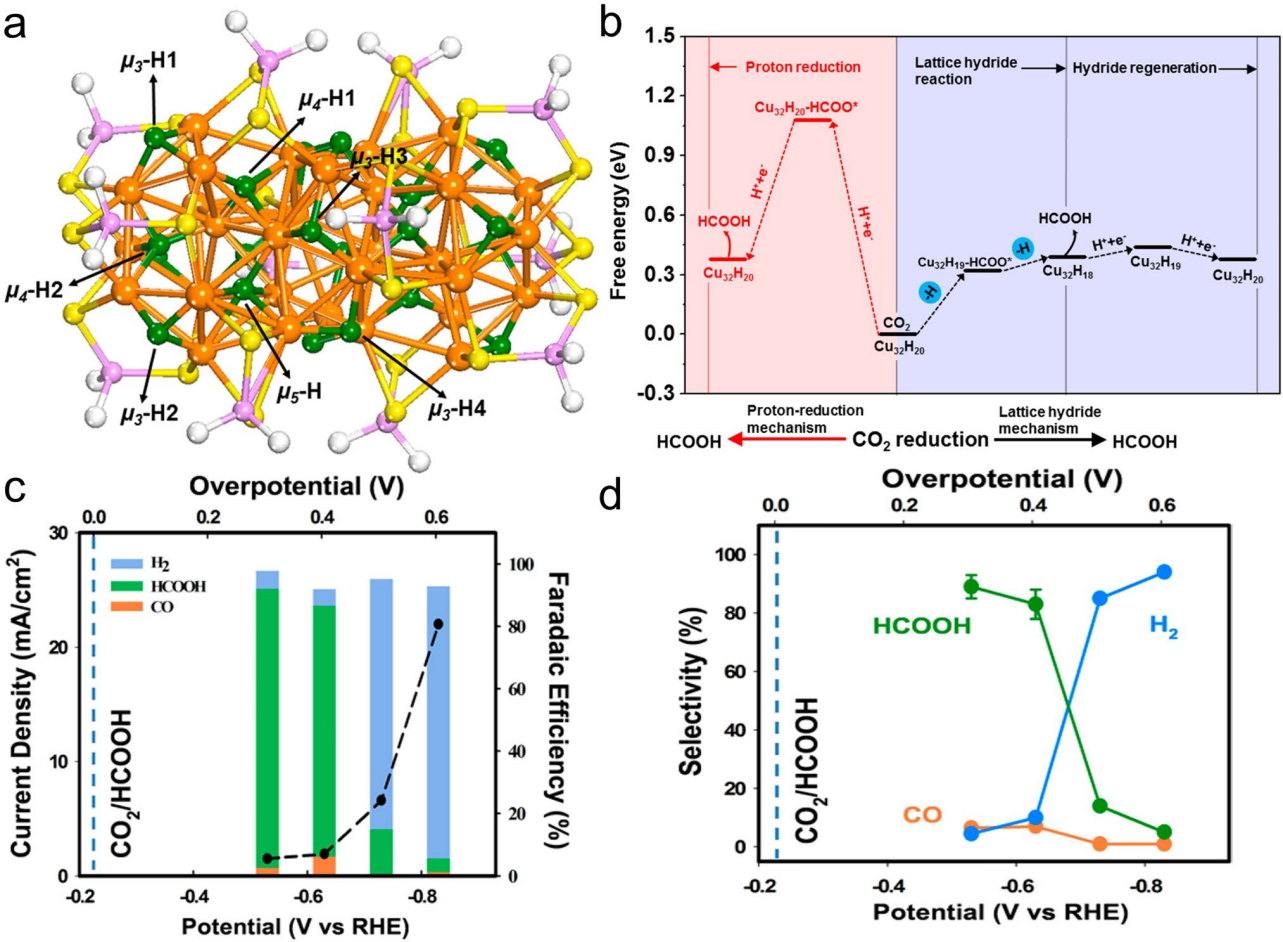
**a** Total structure of Cu_32_H_20_L_12_ nanocluster. Color codes: Cu (orange), S (yellow), hydride (green), H (white), P (purple). **b** CO_2_ electroreduction on Cu_32_H_20_L_12_ to form HCOOH via the proton-reduction channel (left) and the lattice-hydride channel (right). **c** Average current densities (black circles) and cumulative Faradaic efficiencies (stacked bars) obtained at different overpotentials. **d** Product selectivity for H_2_, HCOOH, and CO at different overpotentials. Reproduced with permission from Ref. [51], Copyright 2017 American Chemical Society

In 2017, Tang et al. reported a structurally precise Cu–hydride nanocluster of Cu_32_H_20_L_12_ (L is a dithiophosphate ligand), which offered unique selectivity for eCO_2_RR at low overpotentials [51]. By density functional theory calculations, the authors first predicted that the presence of negatively charged hydride in Cu nanocluster plays a crucial role in determining the selectivity of the products, yielding HCO_2_H over CO at lower overpotentials. The optimized structure of the Cu_32_H_20_L_12_ cluster is shown in Fig. 3a, and the Cu_32_ cluster has a distorted 14-Cu-atom formed hexacapped rhombohedral core sandwiched between two 9-Cu-atom formed triangular cupola fragments. Meanwhile, the 20 hydrides are divided into 12 triply coordinated H (µ_3_-H), 6 tetra-coordinated H (µ_4_-H), and 2 penta-coordinated H (µ_5_-H). For CO_2_ reduction into CO on the Cu_32_H_20_L_12_ cluster, it adopts either the proton-reduction channel or lattice-hydride mechanism (Fig. 3b). It can be clearly noted that the lattice hydride pathway is more favorable, where the rate-determining step is the μ_3_-H1 hydride being transferred to form the Cu_32_H_19_L_12_-HCOO intermediate with a free energy change of 0.32 eV (Fig. 3b right). In contrast, the formation of Cu_32_H_19_L_12_-HCOO in the proton reduction channel requires an energy gap of 1.08 eV (Fig. 3b left). Finally, the authors conducted the eCO_2_RR test of the Cu_32_H_20_L_12_ cluster to verify the theoretical predictions. As shown in Fig. 3c, the average current density becomes significant at the overpotential of 0.3 V and increases with the increasing of the overpotential. H_2_, CO, and HCOOH are the main products, and the cumulative Faradaic efficiency (FE) is over 90%. The product selectivity is illustrated in Fig. 3d. HCO_2_H is the predominant major product in the overpotential window from 0.3 to 0.4 V, and HER dominates at higher overpotentials (from 0.5 to 0.6 V) [51]. These experimental results confirmed the theoretical predictions, and this study showcases that the hydride-containing Cu nanoclusters may offer a unique product selectivity over conventional transition metal nanocatalysts for eCO_2_RR.

**Fig. 4:**
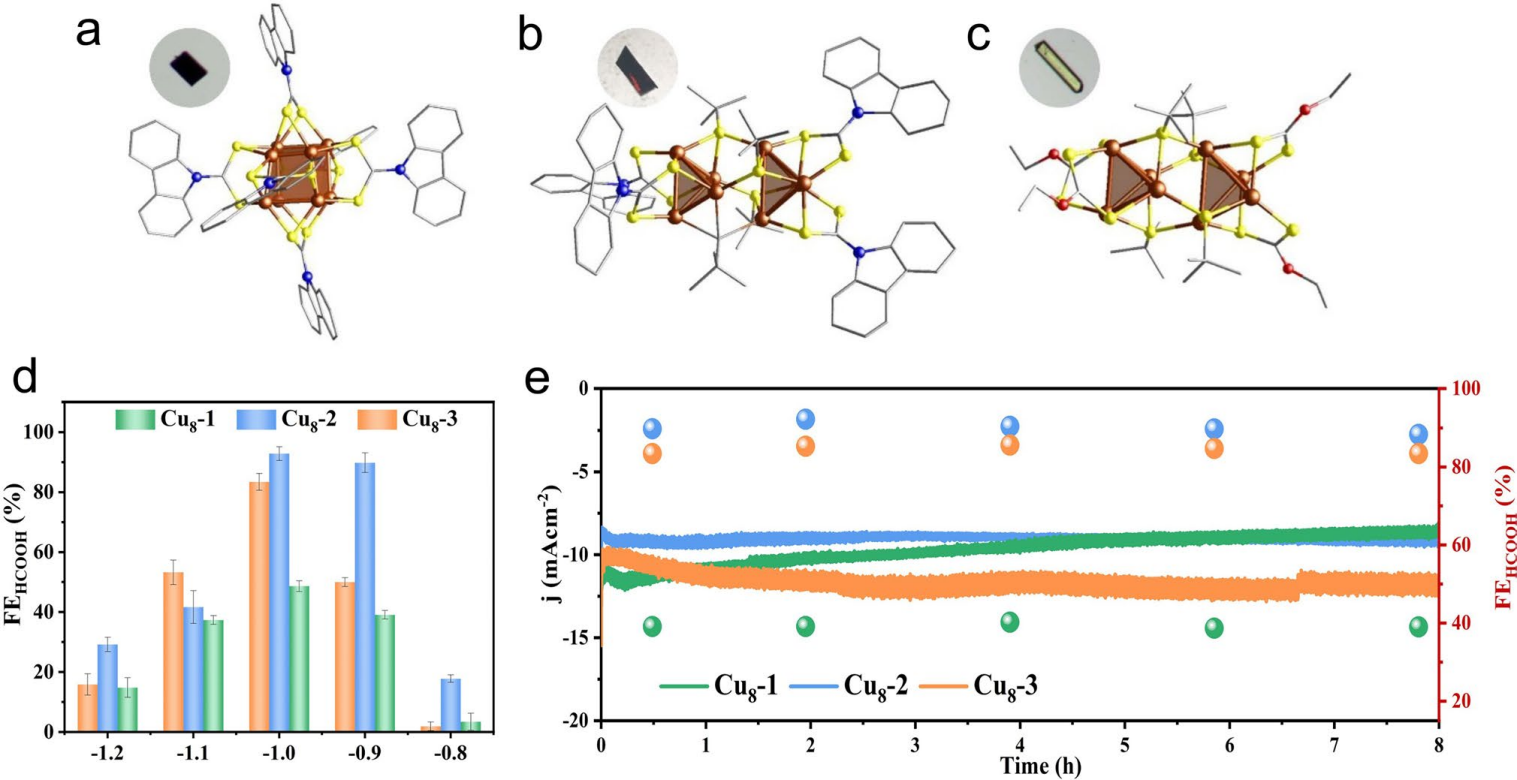
The total structures of **a** Cu_8_-1, **b** Cu_8_-2, and **c** Cu_8_-3. Color codes: Cu (brown), S (yellow), C (gray), O (red), N (blue). Electrocatalytic performances of **d** FE_HCOOH_ of Cu_8_-1, Cu_8_-2, and Cu_8_-3 at different applied potentials. **e** Stability tests of the catalysts for eCO_2_RR (Cu_8_-1 and Cu_8_-2 at -0.9 V, Cu_8_-3 at −1.0 V). Reproduced with permission from Ref. [52], Copyright 2022 Wiley VCH

Furthermore, the core configuration of Cu nanoclusters can be manipulated to further mediate the activity and selectivity of eCO_2_RR. In 2022, the Zang and Wang group reported three novel isomeric Cu_8_-cluster with cores composed of two types of metal kernels (ditetrahedral vs. cubic) and studied the morphological kernel influence on the electrochemical eCO_2_RR at the atomic level [52]. Specifically, three Cu clusters of Cu_8_(H)-(L1)_6_PF_6_, Cu_8_(^*t*^BuS)_4_(L1)_4_, and Cu_8_(^*t*^BuS)_4_(L2)_4_ (Cu_8_-1, Cu_8_-2, and Cu_8_-3, respectively, where L1 = 9*H*-carbazole-9-carbodithioate and L2 = *O*-ethyl carbonodithiolate) were prepared, and the total structures are illustrated in Fig. 4a–c. Cu_8_-1 contains a slightly twisted cubic Cu_8_^8+^ core, while Cu_8_-2 and Cu_8_-3 show the identical di-tetrahedral configurations. In eCO_2_RR test, H_2_, CO, and HCO_2_H were the main products, and the total FE values were over 90% (Fig. 4d). Moreover, the di-tetrahedron-shaped Cu_8_ clusters (Cu_8_-2 and Cu_8_-3) exhibited a higher FEHCOH_2_ and greater selectivity than the cube-shaped Cu_8_ cluster (Cu_8_-1), where Cu_8_-2 demonstrated remarkable HCO_2_H selectivity, manifested by the FEHCO_2_H values of 90% and 92% at −0.9 and −1.0 V, respectively (Fig. 4d). Meanwhile, in the long-term stability test of 8 h, only the current density from Cu_8_-2 remained almost unchanged, indicating robust durability for prolonged operation (Fig. 4e). At last, the free energies of each step for Cu_8_-1 and Cu_8_-2 were calculated. Noteworthily, for the adsorbed *CO_2_ being converted into HCOO*, the di-tetrahedron-shaped Cu_8_-2 cluster (−0.91 eV) has a much lower free energy than the cube-shaped Cu_8_-1 cluster (−0.23 eV). Since the rate determining step is the formation of COOH* to generate HCO_2_H, the Cu_8_-2 cluster is more favorable for HCO_2_H production [52]. This investigation showcases the modulation of activity and selectivity toward CO_2_ electroreduction by tailoring the Cu core, which can probably trigger more endeavors to design tailored Cu based catalysts for eCO_2_RR.

**Fig. 5:**
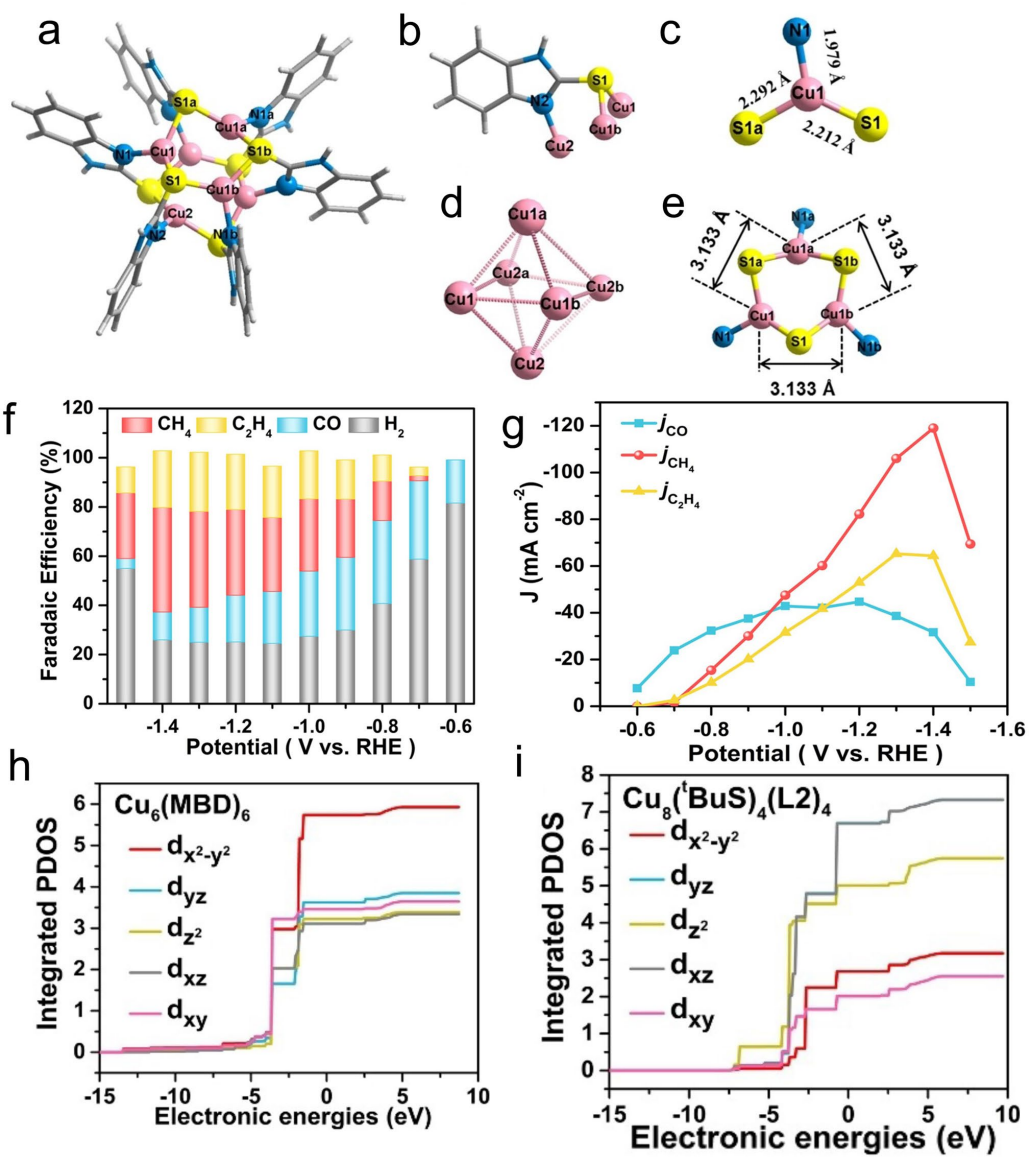
**a–e** Structural analysis of Cu_6_(MBD)_6_ nanocluster. Color codes: pink, Cu; blue, N; yellow, S; deep gray, C; light gray, H. **f** FEs of CH_4_, C_2_H_4_, CO and H_2_ at different potential on Cu_6_(MBD)_6_. **g** Partial current densities of CH_4_, C_2_H_4_, and CO on Cu_6_(MBD)_6_. IPDOS of 3d orbitals: d-density of states vs. the Fermi level projected onto Cu atoms in (**h**) Cu_6_(MBD)_6_ and (**i**) Cu_8_(^t^BuS)_4_(L2)_4_. Reproduced with permission from Ref. [27], Copyright 2023 Wiley VCH

It is worth noting that for most reported atomically precise Cu nanoclusters in the eCO_2_RR, C1 products of CO and HCO_2_H with high selectivity are normally obtained. This is probably due to the Cu active sites are coordination symmetric (typically CuS_3_). Compared to the C1 products of CO/HCO_2_H, the high energy density hydrocarbon products such as CH_4_ and C_2_H_4_ are highly sought as potential fuels [127–130]. To switch the C1 products to more valuable hydrocarbons, Wu et al. recently reported a catalyst based Cu_6_ nanocluster with symmetry-broken CuS_2_N_1_ active sites [27]. The total structure of the as-prepared Cu_6_(MBD)_6_ nanocluster (MBD = 2-mercaptobenzimidazole) is shown in Fig. 5a. Each MBD ligand has the tridentate sites to coordinate three Cu atoms, where the thiolate S atom binds with two Cu atoms and the N atom binds to the other Cu atom (Fig. 5b). Meanwhile, each Cu atom is coordinated by two S atoms and one N atom (Fig. 5c). It is symmetry-broken distorted CuS_2_N_1_ geometry, as the bonding length values are all different (Cu_1_−S_1_ = 2.2118 A, Cu_1_−S_1a_ = 2.2924 A, Cu_1_−N_1_ = 1.9757 A). In addition, six Cu atoms formed a distorted octahedron with the Cu–Cu bonding ranging from 3.083 to 3.254 Å, which might be favorable for C–C coupling (Fig. 5d). There is sufficient space above the distorted CuS_2_N_1_ geometry, which can be accessible for CO_2_ adsorption (Fig. 5e). When using Cu_6_(MBD)_6_ nanocluster as the catalyst, the product distribution is shown in Fig. 5f. When the potential goes from −0.7 to −1.4 V, the FE_CO_ and FE_H2_ gradually decreased; meanwhile, the FEC_2_H_4_ and FEC_2_H_4_ gradually increased (Fig. 5f). The highest FE_CH__4_ reached 42.5% at −1.4 V with a large partial current density of −119.0 mA cm^−2^, with the FEC_2_H_4_ of 23.0% and the partial current density of −64.4 mA cm^−2^ (Fig. 5g). That is, in a wide potential window, CH_4_ and C_2_H_4_ are the dominant products rather than CO. To gain more insights into the advantages of symmetry-breaking at the catalytic sites, in situ Cu K-edge XANES measurement of Cu_6_(MBD)_6_ was conducted, revealing that there is clear electron transfer from Cu_6_(MBD)_6_ to the CO_2_ molecule. Thus, the Cu_6_(MBD)_6_ cluster with asymmetric CuS_2_N_1_ sites has a better CO_2_ activation capacity than the Cu_8_(^t^BuS)_4_(L2)_4_ cluster with the symmetric CuS_3_ sites. The integrated projected density of states (IPDOS) of Cu sites in Cu_6_(MBD)_6_ and Cu_8_(^*t*^BuS)_4_(L2)_4_ were then calculated. For Cu_6_(MBD)_6_, the d_*x*2-*y*2_ orbital of Cu-S_2_N_1_ site has the highest number of electrons close to the Fermi energy level (Fig. 5h), whereas the highest d orbital is Cu d_*xz*_ in CuS_3_ sites of Cu_8_(^*t*^BuS)_4_(L2)_4_ (Fig. 5i). It suggests that the coordination-symmetry breaking significantly affects the highest occupied d-orbital, which plays a critical role in regulating the coordination mode of the reactant and intermediate. When the C atom of CO_2_ is adsorbed onto the Cu_6_(MBD)_6_, the highest occupied d_x2-y2_ orbital can energy-match with the lowest occupied π* orbital of CO_2_ to form a *π*-complex rather than the conventional *σ*-complex, where the *π*-complex can lower the energy barrier of CO_2_ activation. If the O atom of CO_2_ is adsorbed onto the Cu_6_(MBD)_6_, a *σ*-complex is formed, and for Cu_8_(^*t*^BuS)_4_(L2)_4_, a *π*-complex is formed only when the O atom in CO_2_ is the adsorbed atom. Consequently, due to superiority of the C atom adsorbing on Cu_6_(MBD)_6_, the hydrogenation on the less hindered O atom to form the key *COOH intermediate rather than the *OCHO intermediate is more favorable, making the reaction proceed to yield the CH_4_/C_2_H_4_ products [27]. This study highlights the importance of regulating the coordination mode of Cu nanoclusters, which might be a promising direction for designing metal nanoclusters as highly efficient CO_2_ reduction electrocatalysts toward valuable hydrocarbon production.

The large surface area, good catalytic activity and strong capability to suppress HER also enable Cu nanoclusters as potent catalysts for electrochemical nitrate reduction reaction (eNO_3_RR) [131, 132], a process that can turn nitrate contaminant into valuable NH_3_ product. Specifically, NH_3_ is an important feedstock for preparing nitrogen-containing fertilizer, pharmaceuticals, chemicals, and agricultural products, and it is also a valuable green energy carrier [133, 134]. However, the current global production of NH_3_ is largely relying on the energy-intensive and large CO_2_-emissive Haber–Bosch process. The electrochemical N_2_ reduction powered by renewable energy offers a promising approach but it is significantly restricted by the low solubility of N_2_ in water and the extremely high energy required to break the N≡N bond (941 kJ mol^−1^). In stark contrast, nitrate is one of the major contaminants in wastewater and also has much weaker N–O bonding energy (250 kJ mol^−1^). Therefore, developing efficient and stable electrocatalysts for NO_3_RR is imperative for offering a sustainable strategy to produce NH_3_ [135–138]. Recently, atomically precise metal nanoclusters have demonstrated great potential for catalyzing eNO_3_RR, mainly thanks to the strong catalytic capability, and more importantly, the atomic precise structure can provide an ideal model to elucidate the complicated reaction pathway and establish the structure–activity correlation [131, 132, 139]. For instance, our group found that Ag_30_Pd_4_(C_6_H_9_)_26_](BPh_4_)_2_ nanocluster can achieve the Faradaic efficiency of NH_3_ over 90% in eNO_3_RR, where the Ag site is responsible for converting NO_3_^−^ into NO_2_^−^ and the Pd site makes the major contribution to catalyze NO_2_^−^ into NH_3_ hence the whole reaction adopted a tandem catalytic mechanism [93].

**Fig. 6:**
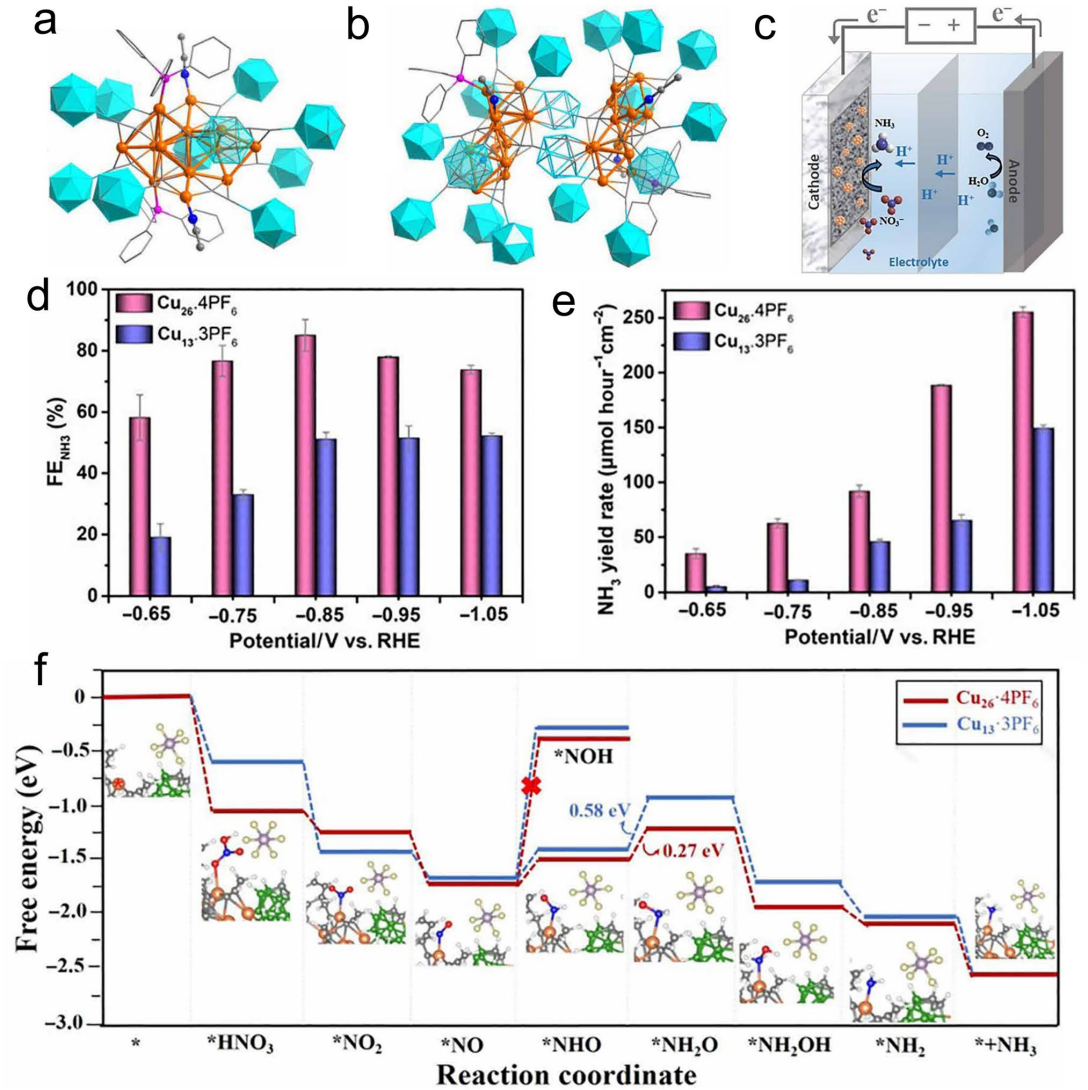
Structure of **a** Cu_13_·3PF_6_ and **b** Cu_26_·4PF_6_·**c** Schematic illustration of NO_3_RR. **d** Comparison of FE_NH3_ at various potentials. **e** Potential dependent NH_3_ yield rate for Cu_13_·3PF_6_ and Cu_26_·4PF_6_. **f** Corresponding adsorption configurations of the reaction intermediates and Gibbs free energy in eNO_3_RR. Cu, orange; F, yellow; P, violet; B, green; C, gray; O, red; N, blue; H, white. Reproduced with permission with Ref [53]. Copyright 2024 American Association for the Advancement of Science

Recently, Wang and Zang et al. utilized bulky carborane-alkynyl ligand to prepare atom-precise monomer Cu_13_·3PF_6_ and bridged dimer Cu_26_·4PF_6_ clusters, and both clusters exhibited remarkable catalytic activity and selectivity in eNO_3_RR [53]. As illustrated in Fig. 6a, Cu_13_·3PF_6_ has a metal skeleton, which could be viewed as two pentagonal bipyramids merged by sharing one equatorial edge but with one vertex of the pentagonal bipyramid being lost. Cu_26_·4PF_6_ is a dimer of Cu_13_·3PF_6_. To be specific, in Cu_26_·4PF_6_, each Cu_13_ monomer loses a carbocycloalkynyl ligand and the adjacent PPh_3_, and the vacated space is occupied by the nido-carboranealkynyl ligands unit of the other monomer via cyclopentadienyl anionic coordination (Fig. 6b). Interestingly, both clusters have fair stability and accessible open metal sites for eNO_3_RR, where eNO_3_RR was carried out in an H-type cell containing 0.1 M KNO_3_ in 0.5 M K_2_SO_4_ medium (Fig. 6c). When the applied potential goes more negatively, the FENH_3_ exhibited a volcano-shape change, and both clusters reached the maximal value at -0.85 V (vs. RHE). The maximal FENH_3_ of Cu_26_·4PF_6_ is 85.1%, much higher than that of Cu_13_·3PF_6_. Meanwhile, at each measured potential, the FE and yield rate of NH_3_ for Cu_26_·4PF_6_ surpass that of Cu_13_·3PF_6_ (Fig. 6d, e), despite the NH_3_ yield rate increasing with the increasing of the applied potential (Fig. 6e). Finally, the authors elucidated the reaction pathway and mechanism with the aid of theoretical simulations and in-situ FTIR spectroscopic study. The free energy diagram in Fig. 6f suggests that Cu_13_·3PF_6_ and Cu_26_·4PF_6_ share the identical reaction pathway (*NO_3_ → *NO_2_ → *NO → *NHO → *NH_2_O → *NH_2_OH → *NH_2_ → *NH_3_). For both clusters, the rate determining step (RDS) is identified as *NHO-to-*NH_2_O, and the energy consumption for Cu_26_·4PF_6_ is much lower than that of Cu_13_·3PF_6_ (0.27 vs. 0.58 eV), indicating Cu_26_·4PF_6_ is more energetically favorable for eNO_3_RR. Moreover, the subsequent *NH_2_O-to-*NH_3_ reduction step is also a downhill process, demonstrating a strong interaction between the key intermediates and the active sites, which can promote the electron transfer and the following hydrogenation steps to accelerate the eNO_3_RR process. This study not only establishes a platform to disclose the structure–activity relationship of Cu nanoclusters for eNO_3_RR, but also provides a feasible strategy to access desirable Cu nanoclusters as efficient eNO_3_RR catalysts [53].

### Cu NCs for Photocatalysis

Solar energy is an inexhaustible natural energy source, and photocatalytic technology can utilize solar energy to realize environmental decontamination and energy conversion, particularly, photocatalytic technology can reduce CO_2_ into valuable hydrocarbon fuels [140, 141]. The principle of photocatalytic reduction of CO_2_ is to use photoexcited semiconductor photocatalysts to produce photogenerated electrons and holes, and the carriers take redox reactions on the surface of the catalyst, reducing CO_2_ into useful chemical raw materials [142, 143]. Compared with other methods, the photocatalytic reduction approach has some unique merits. On one hand, photocatalytic method directly uses solar energy for CO_2_ reduction and transformation, which is safe and pollution-free. On the other hand, the device for photocatalytic reduction is quite simple, and it can operate at very mild conditions, so the whole process is economically feasible [144–147].

Among a variety of photocatalysts, the artificial photosynthesis system using inexhaustible solar energy to simultaneously reduce CO_2_ and oxidize H_2_O to produce valuable chemicals has been attracting more and more research attention [148, 149]. The current widely reported photocatalytic system usually comprises noble-metal-containing photosensitizers and/or organic dyes as electron donors, which are sophisticated and synthetically challenging to be available [150]. Therefore, it is highly desired to prepare a single component photocatalyst with suitable optical band and catalytic active center, that is similar to natural photocatalytic system, which can directly convert CO_2_ and H_2_O into value-added chemicals.

Metal nanoclusters, an emerging type of organic–inorganic hybrid material, composed of an organic layer and metal core, are promising candidates as a single component catalyst to realize the above goal [151, 152]. The ultrasmall size, well-defined configuration, and most importantly, the atomically precise structure can provide a perfect platform to probe the atomical level structure–performance understanding regarding the photocatalysis mechanism [153, 154].

**Fig. 7:**
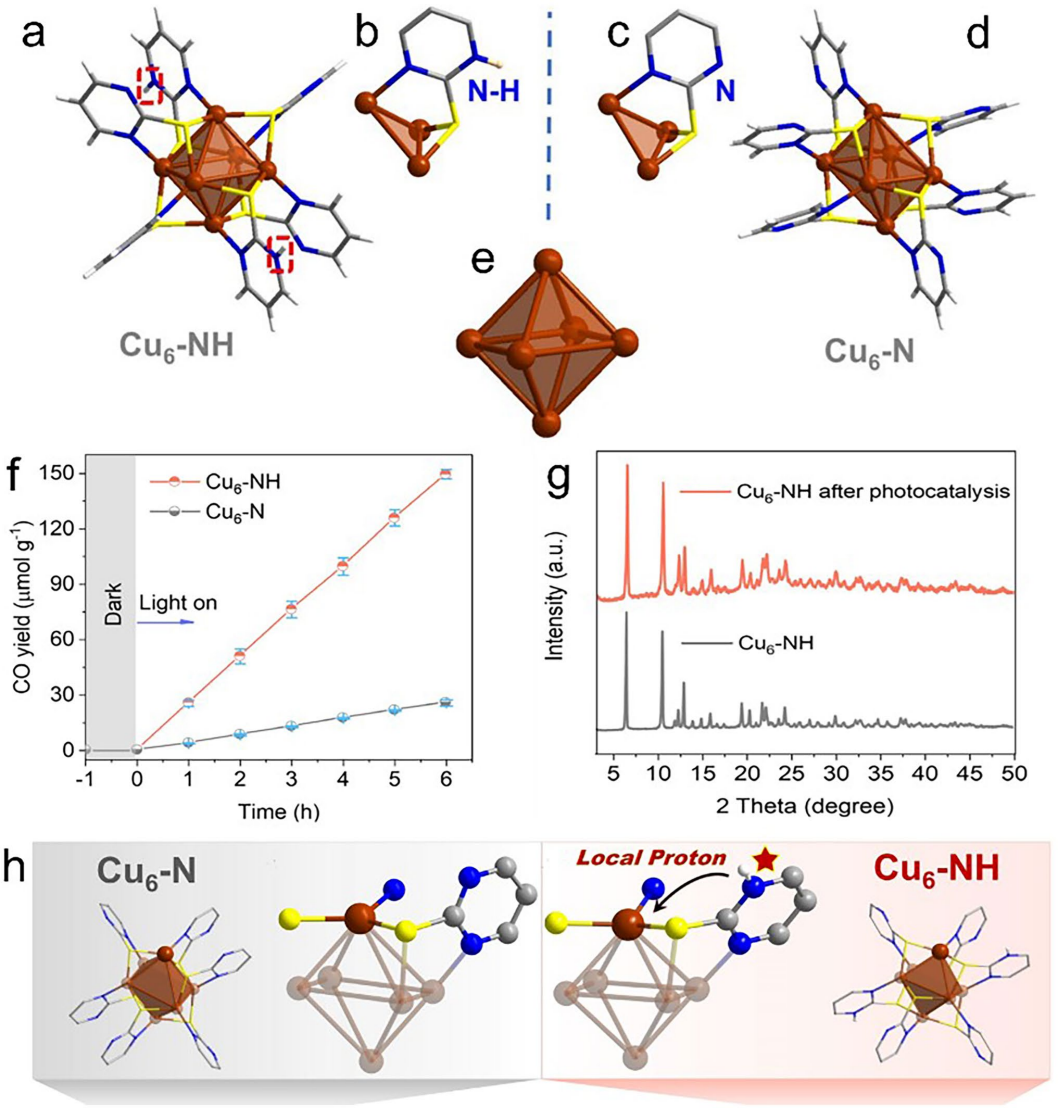
Overall structures of the **a** Cu_6_-NH and **d** Cu_6_-N clusters. Coordination modes in **b** Cu_6_–NH and **c** Cu_6_–N. **e** Cu_6_ framework; The molecule packing diagrams of **f** CO_2_–CO photoreduction performances of Cu_6_–NH and Cu_6_–N. **g** PXRD patterns of Cu_6_–NH before and after 24 h photocatalytic reaction. **h** Different angles showing the Cu_6_ NC structures and the coordination environment around the Cu sites for Cu_6_-N and Cu_6_-NH. Color codes: brown, Cu; yellow, S; gray, C; white, H; blue, N. Reproduced with permission from Ref. [54], Copyright 2023 Wiley–VCH

Recently, the Zang group reported a stable Cu–S–N cluster photocatalyst with local protonated N–H groups, and such cluster can achieve ~ 100% selectivity for CO evolution under visible light. Specifically, two clusters of Cu_6_-NH and Cu_6_-N were prepared [54]. As shown in Fig. 7a, b, Cu_6_-NH cluster possesses a distorted Cu octahedron, and one Cu atom coordinates with one N atom and two S atoms from three ligands. Such configuration is quite identical with the previously reported Cu_6_-N cluster (Fig. 7c, d), except that the ligands in Cu_6_-N are fully deprotonated, despite the two clusters having the same metal kernel (Fig. 7e). Both clusters have considerable absorbance capacities in the wavelength region of 400–550 nm, and comparable band gap energies (2.36 and 2.39 eV for Cu_6_-N and Cu_6_-NH, respectively). However, their visible-light-driven photoreduction capability of CO_2_ showed some significant difference. Both clusters exhibited exceptionally high selectivity of CO with over 99%, but the CO evolution rate is much higher for Cu_6_-NH than that of Cu_6_-N (Fig. 7f). Furthermore, the Cu_6_-NH photocatalyst demonstrated superior catalytic stability, as no detectable change is observed in the XRD patterns before and after the photocatalytic test (Fig. 7g). Finally, they conducted DFT calculations to elucidate the underlying physical origin, and found that, in the rate-determining step of forming *COOH, the Cu_6_-NH cluster has a much lower energy barrier. This is due to the structural difference, that is, the presence of deprotonated pyrimidine N in Cu_6_-N and protonated pyrimidine N in Cu_6_-NH (Fig. 7h). The protonated pyrimidine N atoms in Cu_6_-NH acted as a proton relay station to provide a local proton, hence facilitating the proton coupling process, leading to enhanced photocatalytic efficiency [54]. This study highlights the great promise of using Cu nanoclusters as photosensitive semiconductors for photoreduction of CO_2_.

The precise structure of Cu nanoclusters allows the in-depth study of structure–property relationships in photocatalysis at the atomical level. Moreover, isomeric Cu nanoclusters can make the comparative structure–property relationship study feasible, as they have minimal structural difference. Isomeric Cu nanoclusters usually originate from the chiral isomerism, the structural isomerism, but the quasi-structural isomerism has been ignored for a long time. The quasi-structural isomeric Cu nanocluster usually displays comparative structural features, e.g., core geometric configuration, surface spatial arrangement.

**Fig. 8:**
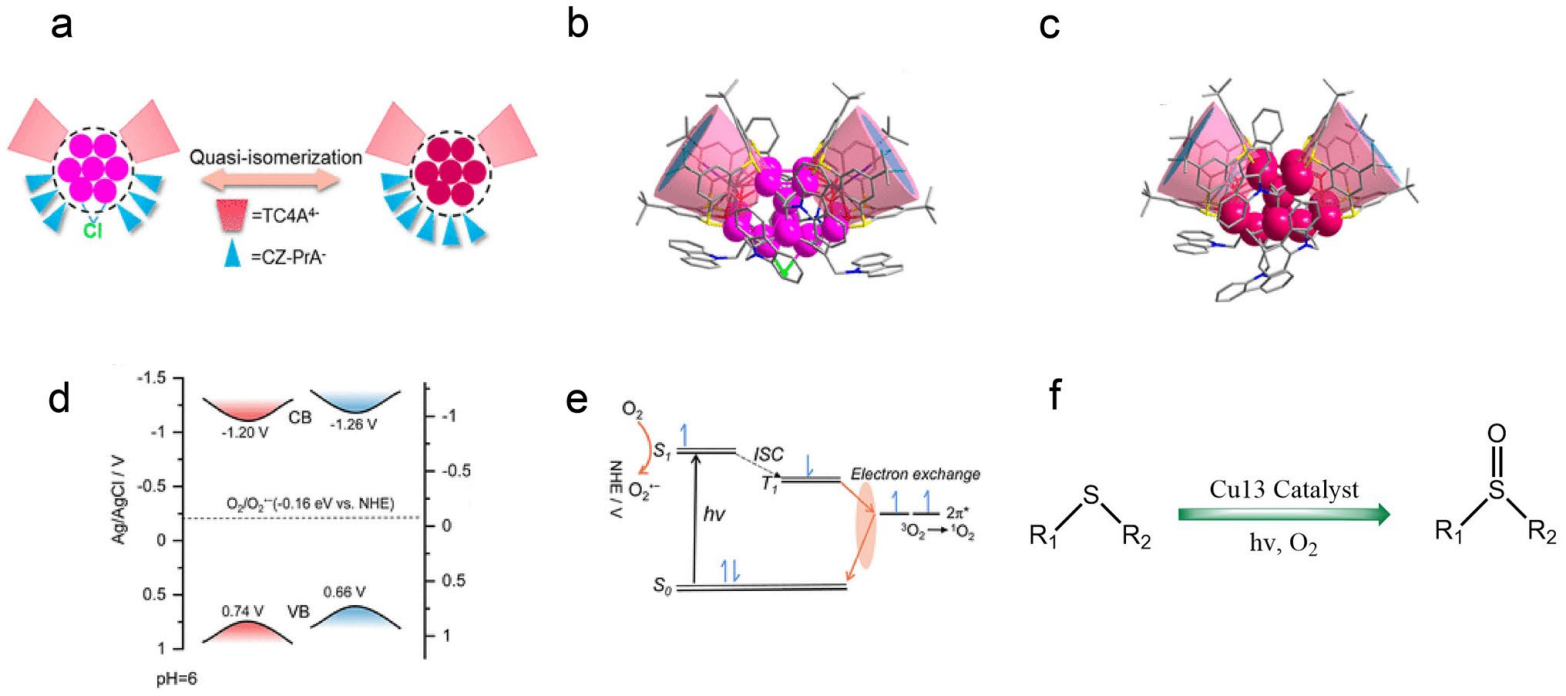
**a** Schematic demonstration of the quasi-isomerization. **b, c** Side views of the molecular structures of Cu13a and Cu13b. **d** Schematic diagram for the band structures of Cu13a and Cu13b. **e** Mechanism of Cu13 cluster and ^3^O_2_ for ^1^O_2_ and O_2_^•−^ photogeneration. **f** Oxidation of sulfides catalyzed by Cu_13_ nanoclusters. Color labels: red and purple, Cu; cyan, Na; gray, C; red, O; yellow, S; blue, N; green, Cl. Reproduced with permission from Ref. [49], Copyright 2022 American Chemical Society

In 2022, the Sun group reported two quasi-structurally isomeric Cu_13_ nanoclusters with highly similar kernel and different spatial arrangements of peripheral ligands (Fig. 8a) [49]. Both Cu13a and Cu13b clusters have a highly similar Cu_13_ kernel but different degrees of distortion (Fig. 8b, c). The formation of these two Cu cluster isomers is governed by the exotic chlorine ion, and also due to the charge transfer from Cl^−^ to Cu core, Cu13a presented lower superoxide radical (O_2_^•−^) yield and higher singlet oxygen (^1^O_2_) compared to that of Cu13b. The conduction band minimum (CBM) is −1.20 and −1.26 V for Cu13a and Cu13b, respectively, so through the band gap value, the valence band maximum (VBM) is calculated as 0.74 and 0.66 V for Cu13a and Cu13b, respectively (Fig. 8d). More negative CBM value drives Cu13b more energetically feasible for O_2_^•−^, while for Cu13a, the larger band gap favors the exchange of an electron with ^3^O_2_, also more efficient for ^1^O_2_ generation (Fig. 8e). Consequently, the two clusters demonstrated different performance particularly in selectivity in sulfides oxidation into sulfoxides. As summarized in Fig. 8f, despite methyl sulfide can be transformed into dimethyl sulfoxide by two clusters with nearly identical high conversion rates (near unity), Cu13a showed a certainly higher degree of enhancement than that of Cu13b [49]. This work provides a new pathway for boosting the photocatalytic selectivity of Cu nanoclusters.

In modern synthesis, bond-forming reactions are critical for generating new products, particularly, bond-forming reactions at mild conditions can find versatile applications in fine chemical synthesis, new drug discovery, and sustainable production of the industrial precursor. To that end, visible light photocatalysis is more advantageous as compared to thermal catalysis, however, developing high-efficiency, stable catalyst is the key [155]. Cu-based photocatalysis has shown great promise as an inexpensive and attractive method, compared to the expensive metal-complex and less stable organic dyes [156, 157]. Nevertheless, the cross-coupling reactions of aryl bromides and iodides are relatively easy to realize, but the cross-coupling reactions of aryl chloride remained extremely challenging.

**Fig. 9:**
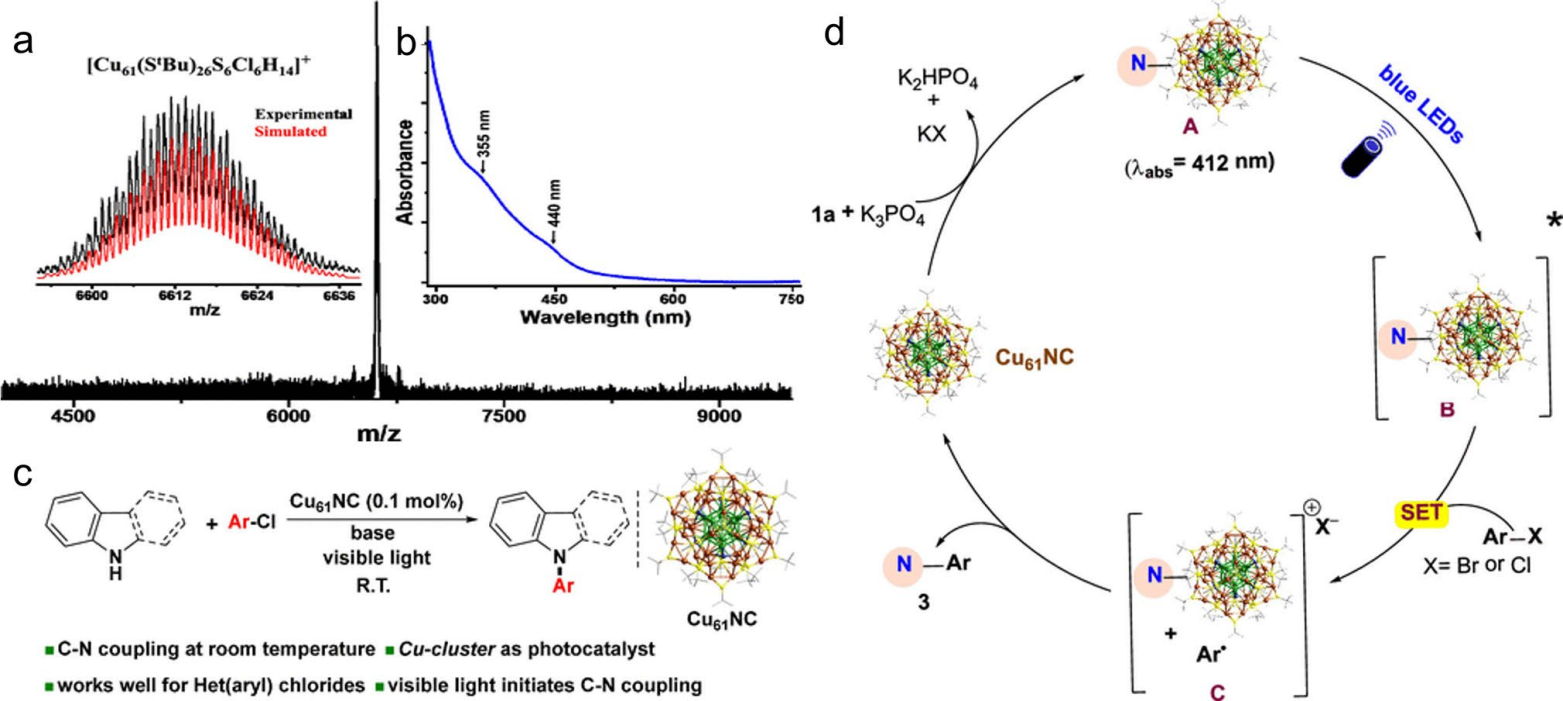
ESI MS spectrum of Cu_61_ NC. The molecular ion peak is at m/z of 6613.5. Insets: **a** Comparison of experimental and simulated isotopic patterns. **b** UV–Vis absorbance spectrum showing two bands at 355 and 440 nm. **c** Cu_61_ NC catalyzed Ullmann C–N Coupling. **d** Proposed reaction mechanism. Reproduced with permission from Ref. [55], Copyright 2022 American Chemical Society

In 2022, the Bakr and Rueping team reported a [Cu_61_(S^t^Bu)_26_S_6_Cl_6_H_14_] nanocluster (Cu_61_NC)-based catalyst that can enable C-N bond-forming reaction of aryl chlorides under visible-light irradiation at ambient conditions [55]. As shown in Fig. 9a, the pronounced molecular ion is observed, and the experimental pattern agrees well with the simulated pattern. Figure 9b illustrates that it has two characteristic bands, and the crystal structure can be found inset. In the presence of base, at room temperature, the Cu_61_ NC cluster can catalyze the Ullman reaction for het(aryl) very well (Fig. 9c). The authors also proposed a plausible mechanism (Fig. 9d). Specifically, Cu NC reacts with the base forming CuNC-amine complex A first, then blue LED irradiation leads to the formation of complex B. After a SET process to give an electron to aryl chloride, the oxidized CuNC-Nu complex, a halide anion, and an arene radical were generated, evidenced by the fluorescence and quenching lifetime tests. After that, the radical attacks the CuNC-Nu complex, delivering the C–N bond formation and recovering the Cu_61_ clusters [55]. This study introduces atomically precise Cu clusters as a new family of photocatalysts for bond-forming reactions under mild conditions.

### Cu NCs for Organic Reactions

#### Click Reaction

The perfect monodisperse and atomically precise nature of molecular metal nanoclusters can facilitate the development of detailed structure–activity relationships [158–160]. Using these atomically precise metal nanoclusters as catalysts can uncover new insights, but some of them may not be suited for this purpose. Specifically, a vast majority of these metal nanoclusters are protected by a shell of thiolate ligands [1, 152]. These ligands can impart the clusters significant thermal and chemical stability, but they can also block some active sites and hence must be partially removed before catalysis can occur [161]. Meanwhile, the strong bonding strength of the metal-sulfur bond makes it hard to remove completely [161]. However, the harsh reaction conditions often lead to the significant structure or nuclearity change of the metal nanoclusters, making the structure–activity relationship unreliable. To address this issue, the researchers switched to other ligands, which do not require harsh pretreatment for activation. To that end, alkynyl molecules, phosphine ligand, halogen, hydride, and their mixed combination have been widely utilized to prepare atomically precise metal nanoclusters including Cu nanoclusters [56, 72, 73, 162].

**Fig. 10:**
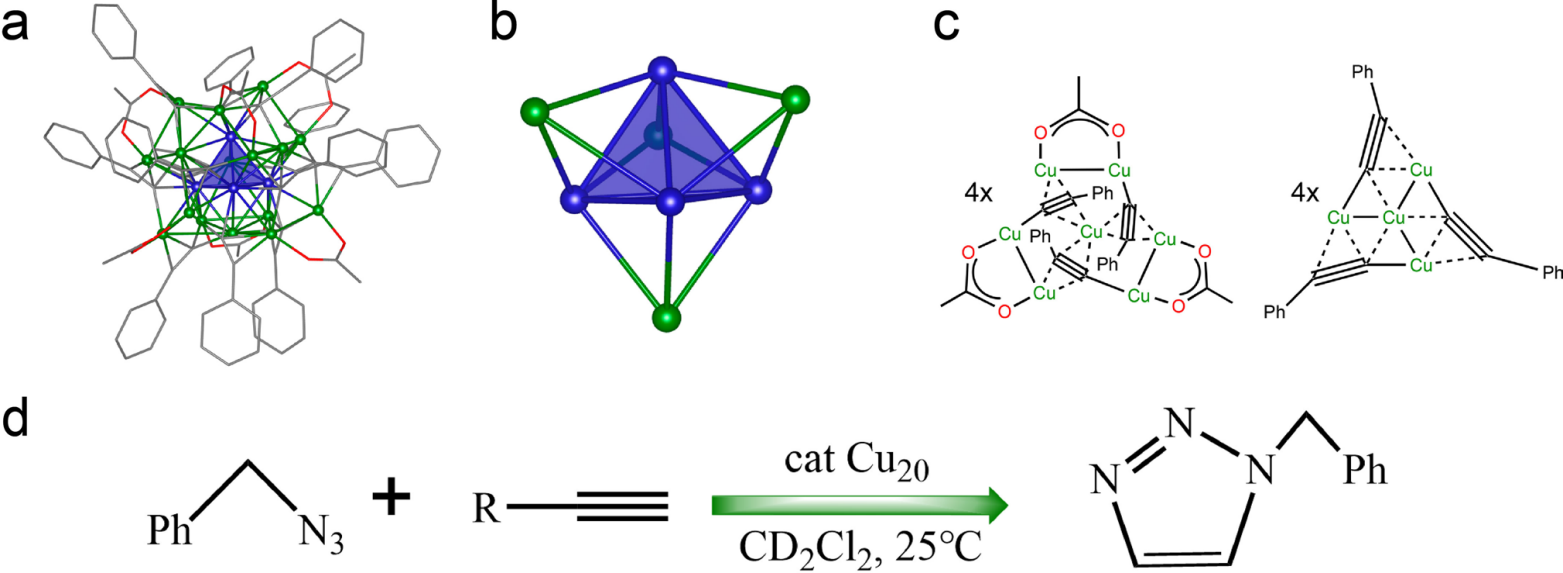
**a** [Cu_20_(C≡CPh)_12_(OAc)_6_]·C_4_H_8_O. **b** Tetrahedral Cu_4_^2+^ core (blue) and face-capping Cu atoms (green). **c** Ligand binding modes. **d** Cycloadditions catalyzed by Cu_20_ nanoclusters. Color legend: Cu = blue, green; C = gray; O = red. Reproduced with permission from Ref. [36], Copyright 2018 American Chemical Society

In 2018, the Hayton group reported the synthesis of [Cu_20_(C≡CPh)_12_(OAc)_6_] cluster and its catalytic application in Click reaction [36]. Figure 10a shows the crystal structure of the Cu_20_ cluster, which has 4 THF molecules incorporated into the crystal lattice as solvates. It has a tetrahedral [Cu_4_]^2+^ core (Fig. 10b), which is encapsulated by a [Cu_16_(C≡CPh)_12_(OAc)_6_]^2−^ shell (Fig. 10c). There are 12 acetylide ligands in the [Cu_16_(C≡CPh)_12_(OAc)_6_]^2−^ shell distributed by 4 [cyclo-Cu(C≡CPh)]_3_ units, which are located at the vertices of a tetrahedron, meanwhile, there are six acetate ligands situated at the edges of the tetrahedron (Fig. 10c). Then the Cu_20_ cluster was immobilized on dry, partially dehydroxylated silica, and the supported cluster catalyst displayed excellent performance toward the Click reaction of cycloadditions between benzyl azide and terminal alkynes. As demonstrated in Fig. 10d, the catalyst is effective for various terminal alkynes without harsh pre-treatment and even with a rather low cluster loading of 0.05 mol%. More importantly, X-ray absorption near edge structure (XANES) test after the catalytic test shows that the cluster undergoes no significant nuclearity changes under click reaction conditions [36]. It also indicates that the Cu species mainly kept as Cu(0) and Cu(I) in the cluster during the reaction process, where the Cu(I) species is well known to be the active site for click reaction. Great promises of using atomically precise Cu nanoclusters to examine the structure/activity relationship have been demonstrated in this study, especially for the non-thiolate protected Cu nanoclusters.

In nanocatalytic regime, elucidating single-atom effect of the nanocatalyst is interesting yet extremely challenging, as one single-atom modification can cause appreciable change to the overall particle’s structure, not to mention that, the size polydispersity and ambiguous surface structure of the metal nanoparticle [163]. Nevertheless, atomically precise metal nanoclusters have created great opportunities to tailor the chemical structure at the atomic level.

**Fig. 11:**
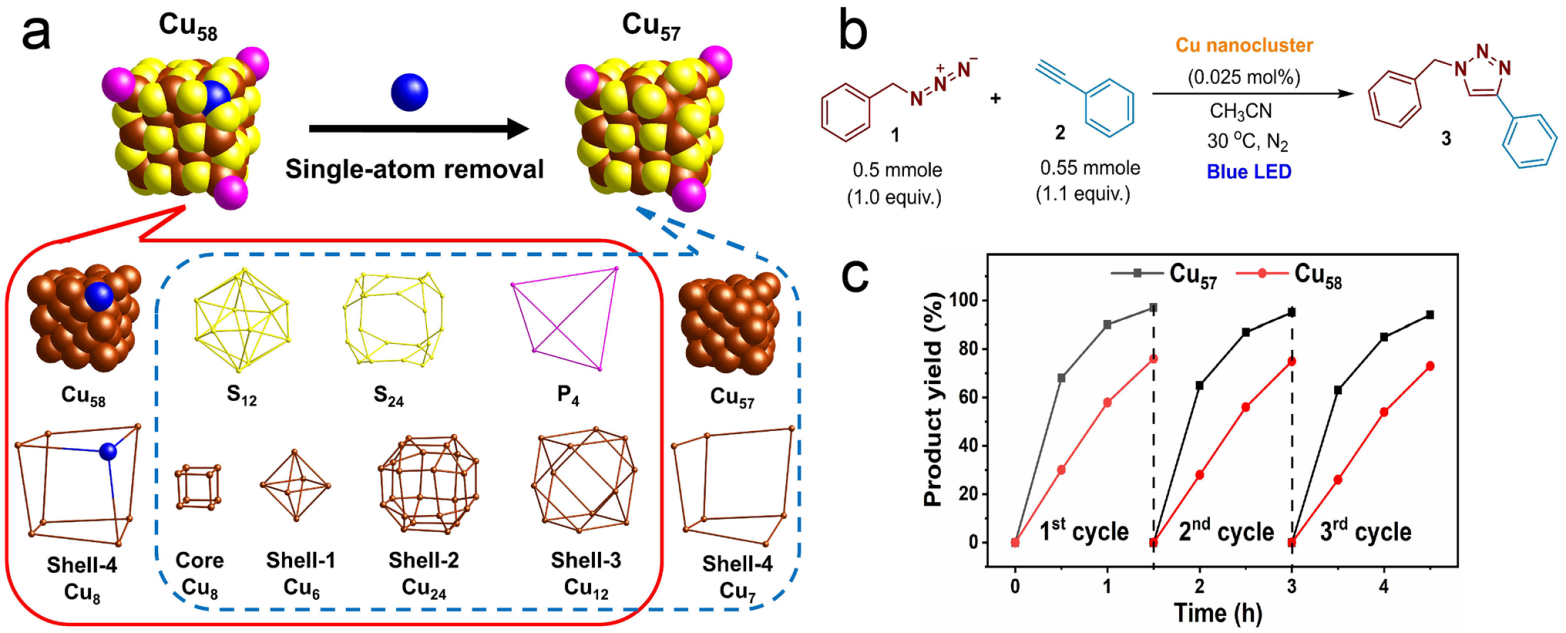
**a** Crystal structure and dissection of Cu_58_ and Cu_57_. **b** Cycloaddition between phenylacetylene and benzyl azide using Cu_58_ and Cu_57_ as catalyst under visible light. **c** Time-dependent product yields of Cu_58_-catalyzed AAC under visible light. Brown and blue: Cu; yellow: S; pink: P. Reproduced with permission from Ref. [57], Copyright 2023 Wiley VCH

Recently, the Bakr and Rueping team reported the synthesis of [Cu_58_H_20_PET_36_(PPh_3_)_4_]^2+^ (Cu_58_) and its analogue of [Cu_57_H_20_PET_36_(PPh_3_)_4_]^+^ (Cu_57_) with one surface Cu atom removed, and Cu_57_ showed much higher catalytic activity toward [3 + 2] azide-alkyne cycloaddition (AAC, click reaction) than that of Cu_58_ [57]. As illustrated in Fig. 11a, Cu_58_ has a multishell architecture, and the metal skeleton possesses five concentric shells of Cu_8_@Cu_6_@Cu_24_@Cu_12_@Cu_8_. Meanwhile, the ligand layer in Cu_58_ consists of a P_4_ tetrahedron, an S_12_ icosahedron, and an S_24_ truncated cube. Cu_57_ has nearly the identical metal kernel structure except one Cu atom was removed on shell-4. Subsequently, the two nanoclusters were employed as catalyst for the click reaction. With the 0.025 mol% of Cu_57_ or Cu_58_ stoichiometric ratio, and in the presence of 1.5 h light irradiation, the product yields of 97% and 77% were achieved for Cu_57_ and Cu_58_, respectively (Fig. 11b). In addition, both clusters showed good catalytic stability, as no obvious product yield decline was observed after three catalytic cycles (Fig. 11c). Overall, Cu_57_ performed more efficiently than Cu_58_ in the click reaction, and one single atom removal made great difference in the nanoclusters’ catalytic activity [57]. This work may open a new avenue for the nanoparticles’ catalytic design by targeted isostructural single-atom manipulation.

#### C–C Coupling Reaction

The C–C bond formation is a widely employed transformation in natural product synthesis, pharmaceutical synthesis, medicinal chemistry, and preparation of functional materials. So far, various catalytic methods have been developed for this important cross-coupling reaction. Among that, Sonogashira cross-coupling reaction is one of the most important and widespread used *sp*^2^–*sp*^2^ carbon–carbon bond formation reactions in organic synthesis, which is usually catalyzed by Pd and other transition metals [157, 164]. It can realize the coupling of aryl or vinyl halides with terminal acetylenes, but most of the catalysts suffer from that the undesired alkyne homocoupling side reaction is extremely difficult to suppress.

**Fig. 12:**
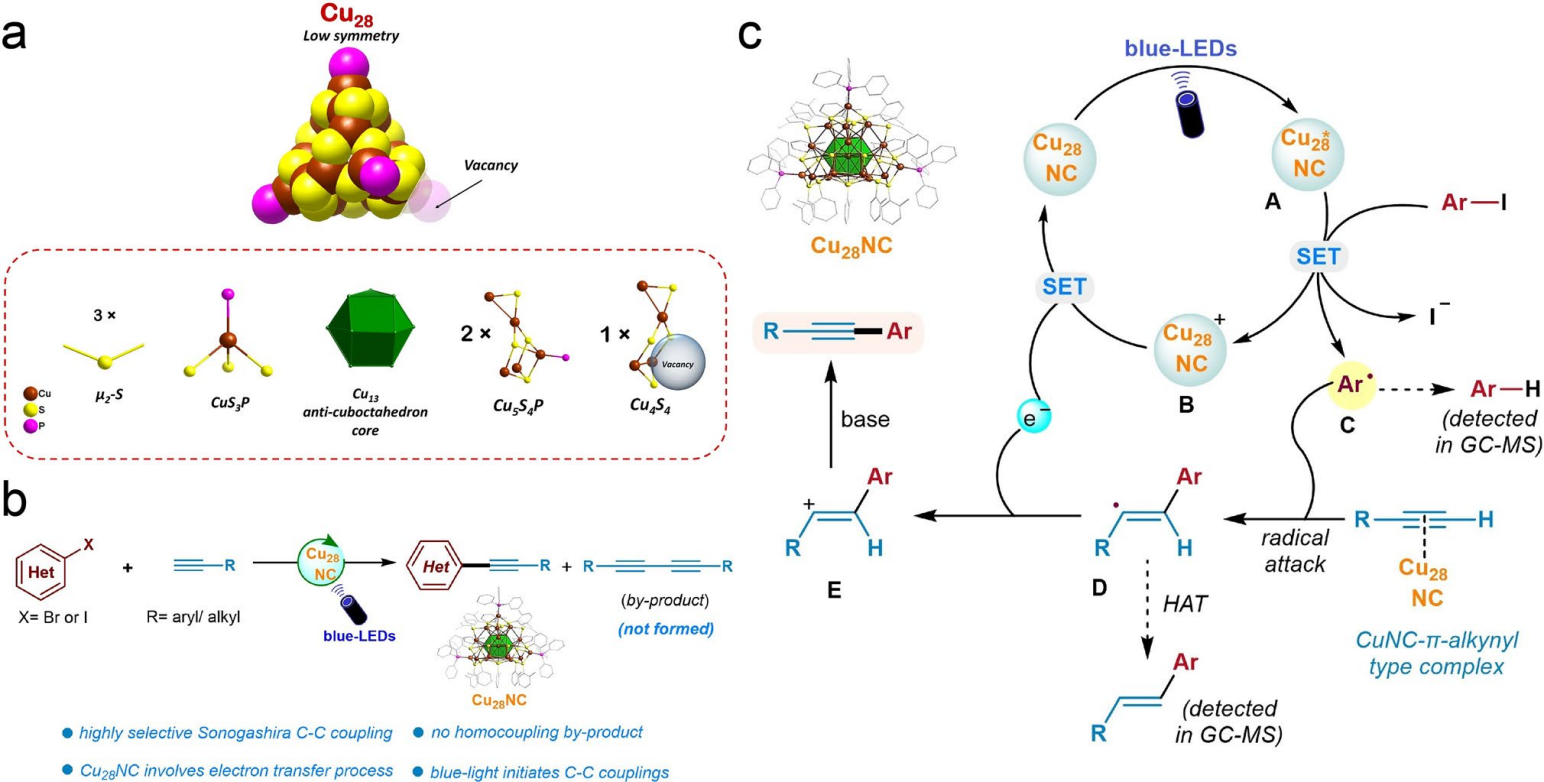
**a** Structure of Cu_28_ nanocluster. **b** Sonogashira C–C coupling reaction. **c** Proposed reaction mechanism. Reproduced with permission from Ref. [58], Copyright 2023 Wiley VCH

In 2023, the Rueping and Bakr group reported the [Cu_28_H_10_(C_7_H_7_S)_18_(TPP)_3_] cluster (Cu_28_; C_7_H_7_S: o-thiocresol; TPP: triphenylphosphine) with a defined defect for Sonogashira C–C coupling reaction. The skeleton structure of Cu_28_ is shown in Fig. 12a, and it features a centered Cu_13_ anti-cuboctahedron core, like the Cu_25_, Ag_19_, and Ag_25_ nanoclusters, and Cu_28_ also has a unique cage-like Cu_15_S_18_P_3_ shell. Interestingly, Cu_28_ is a defective nanocluster which contains a surface vacancy, as one Cu atom is missing in one tetrahedron’s vertex, and this vertex is expected to connect with a phosphine ligand. Subsequently, Cu_28_ was examined as a catalyst for Sonogashira C–C coupling reaction. Upon UV-LEDs irradiation, the desired C–C coupling product with a high yield of 82% was acquired without the byproduct of homocoupling reaction (Fig. 12b) [58]. The authors then conducted the mechanistic control studies and proposed the tentative reaction mechanism (Fig. 12c). Upon blue-light irradiation, Cu_28_ was photoexcited (**A**), and it can induce a single electron transfer (SET) process with aryl iodides to yield the Ar-radical (**C**) and oxidized Cu_28_^+^ (**B**). The Ar-radical then attacks the alkyne C–C triple bond to generate vinylic type C-radical intermediate (**D)**. Another single electron transfer process to B can regenerate the Cu_28_ photocatalyst. Note that the C–C triple bond can be activated by Cu_28_ through forming a Cu NC-*π*-alkynyl type complex hence easily being attacked by the Ar-radical. The authors detected a little stilbene-type byproduct in GC–MS measurement generated by a hydrogen atom transfer (HAT) process. Finally, the base-induced deprotonation of vinyl cation E leads to the desired Sonogashira C–C coupled product [58]. Upon the photoexcitation of blue-LEDs, the oxidation state of the Cu_28_ cluster undergoes a transient transition from 0 to I, form [Cu_28_]^+^ NC, while after the second single electron transfer (SET) process, it returns to the ground state of Cu_28_.The results demonstrate that Cu_28_ is an active photocatalyst enables the SET process with aryl iodides to produce Ar-radical which attacks the C–C triple bond, totally preventing the formation of homocoupling product. This investigation paves a way to acquire Cu NCs with defined surface defects as active sites, and more importantly, these defective Cu NCs can serve as model systems to provide profound understanding on defect effect for heterogeneous catalysis.

**Fig. 13:**
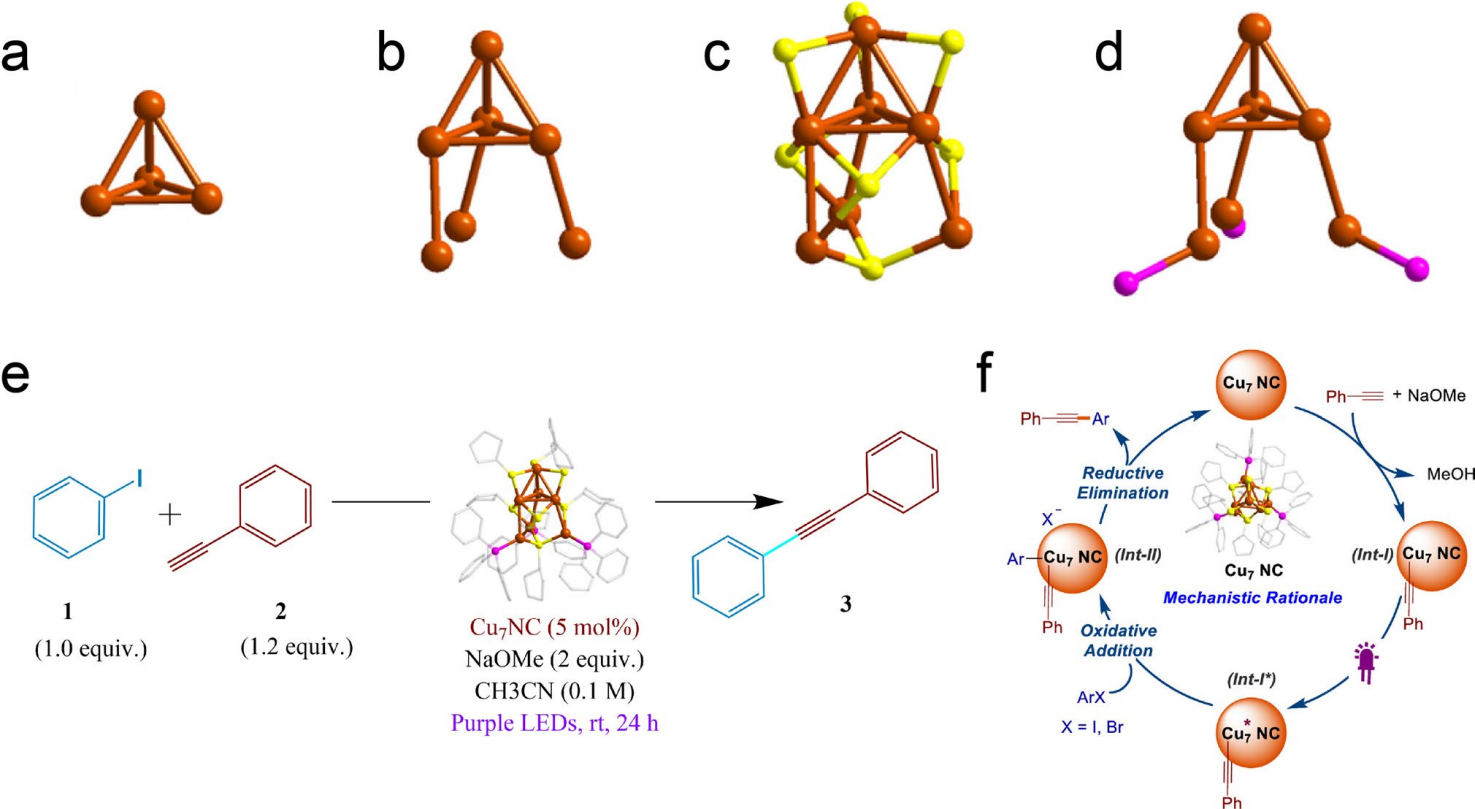
**a** Cu_4_ head. **b** Three Cu atoms connected with the Cu_4_ head. **c** S-Cu binding configuration and **d** P-Cu binding configuration in the Cu_7_ core. **e** Reaction optimization of C_sp2_–C_sp_ cross-coupling in the presence of Cu_7_ NC and **f** proposed reaction mechanism. Cu, brown; S, yellow; and P, magenta. Reproduced with permission [59], Copyright 2024 American Chemical Society

Recently, Biswas et al. reported a hydride-free [Cu_7_(SC_5_H_9_)_7_(PPh_3_)_3_] (Cu_7_) nanocluster (NC), which demonstrated remarkable specificity in a photoinduced C–C coupling reaction [59]. The Cu_7_ NC has a metal core with 4 Cu atoms forming a tetrahedron (Fig. 13a), and three Cu atoms are connected with the bottom face of the tetrahedron with another three Cu atoms (Fig. 13b). The metal–ligand binding configurations are shown in Fig. 13c, d, where the thiolate ligands mainly adopt the bridging modes, and three P atoms connected with three Cu atoms directly. Interestingly, the Cu_7_ NC exhibits intriguing photoluminescence (PL). Upon exciting the cluster at 395 nm, a robust PL emission is observed at room temperature with an emission peak occurring at 436 nm. Such outstanding photophysical properties inspired the authors to explore the Cu_7_ NC as catalyst for photoinduced C–C bond formation reaction. Under purple light irradiation from LEDs, the Cu_7_ NC (5 mol% Cu) was able to catalyze the C_sp2_-C_sp_ coupling reaction between phenyl iodide and phenyl acetylene to generate the desired product with high yield (Fig. 13e). With the aid of control experiments and DFT calculations, the authors also proposed tentative reaction mechanism (Fig. 13f). Initially, the Cu active site in Cu_7_ NC binds to alkyne to form an intermediate of **Int-I** in the presence of MeONa. Subsequently, **Int-I** is photoexcited to generate Int-I*, which engages the oxidative addition step to yield the intermediate of **Int-II**. The following reductive elimination of **Int-II** produces the final product, along with the release of the Cu_7_ NC. In this mechanistic rationale cycle, the oxidative addition step is identified as the rate-determining step [59]. This work provides some mechanistic insights of using atomically precise Cu nanoclusters for C–C coupling reactions, and underscore the great potential of hydride-free Cu nanoclusters for catalysis.

#### A^3^ Coupling Reaction

To forge new C–C and C–N bonds, three-component dehydrogenative coupling reaction represents a new practical methodology. Yet, to realize all-in-one three-component dehydrogenative coupling in a single catalytic system remains very challenging [165]. For instance, to fulfill the efficient synthesis of propargylamines including C1-propargylamines, Cu compounds are the most widely recognized catalysts to implement the A^3^ coupling reactions or redox-A^3^ coupling reactions, but the reaction conditions are rather harsh [166]. Therefore, a Cu-based catalytic system with high efficiency and regio-selectivity with broad substrate scope working under mild conditions is highly desirable.

**Fig. 14:**
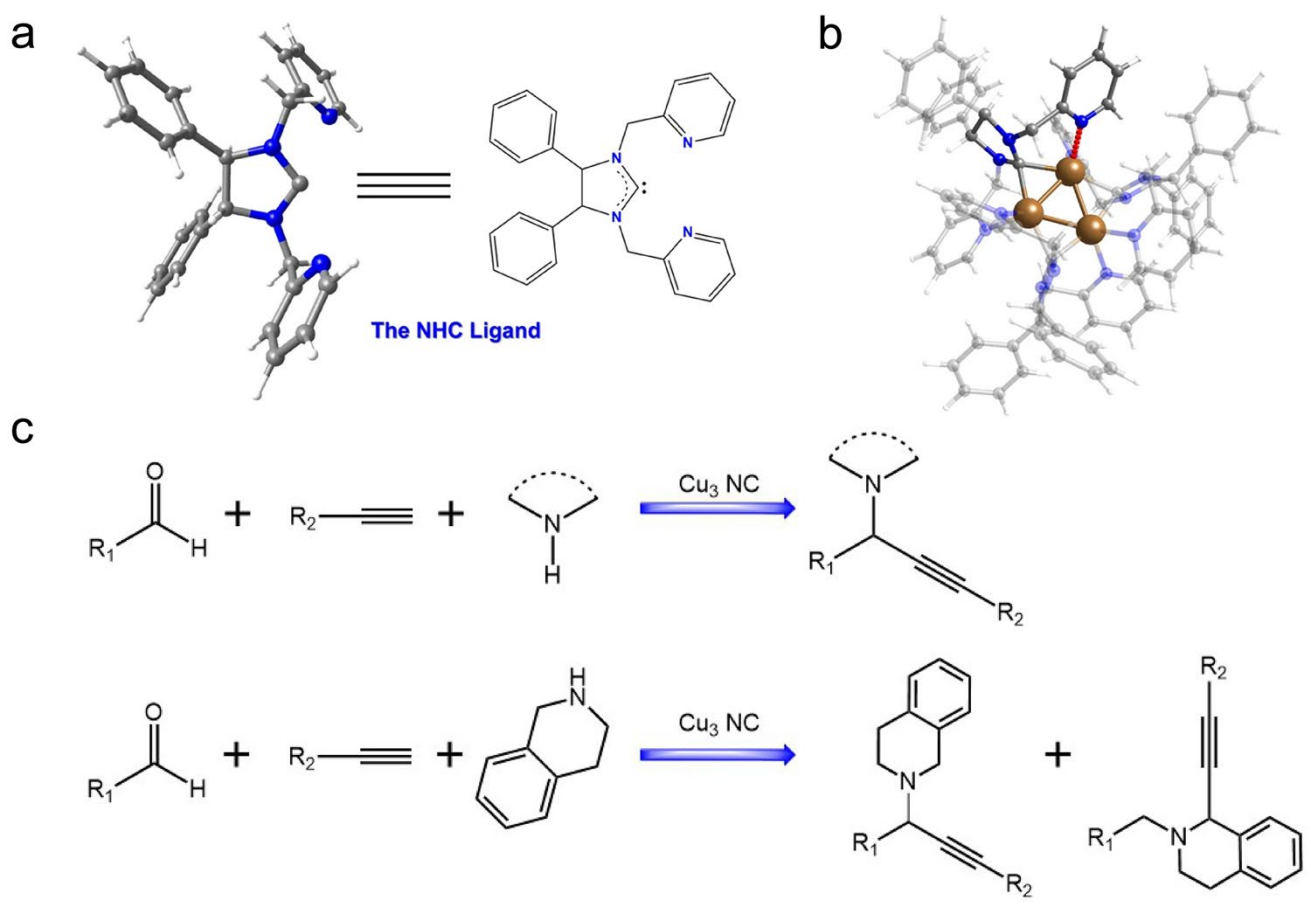
**a** Structure of the N-heterocyclic carbene (NHC) ligand. **b** Total structure of the Cu_3_NC. **c** Cu_3_NC catalyzed both the A^3^ coupling reaction and the redox-A^3^ coupling reaction. R_1_, R_2_, R_3_ and R represent kinds of functional group. Reproduced with permission from Ref. [160], Copyright 2023 Nature Publishing Group

Recently, the Zang group reported a tridentate N-heterocyclic carbene ligand stabilized [Cu_3_(NHC)_3_(PF_6_)_3_] (Cu_3_NC) cluster for both A^3^ coupling reactions and redox-A^3^ coupling reactions [60]. The crystal structure of the NHC ligand and Cu_3_NC are shown in Fig. 14a, b, respectively, where the NHC ligand endows the Cu_3_NC with dual attributes of flexibility and rigidity. On one hand, the stable Cu–C and Cu–N bonds between the NHC ligand shell and the metal core favors the stability of the Cu_3_NC. On the other hand, the pyridine of the N‑heterocyclic carbene has somewhat dynamic balance between the aliphatic amines and the pyridine to protect the catalytic centers and prevent the Cu_3_NC deactivation. Such dynamic balance endows Cu_3_NC with flexible features. Consequently, high activity and high regioselectivity can be achieved in all-in-one A^3^ coupling reactions with inert substrates at room temperature (Fig. 14c), as manifested by 71 examples of A^3^ coupling reactions and 14 examples of redox A^3^ coupling reaction, both up to 99% yield [60]. The following mechanistic and control experimental tests demonstrated that the remarkable catalytic performance originates from the flexible and rigid dual attributes of the Cu_3_NC.

**Fig. 15:**
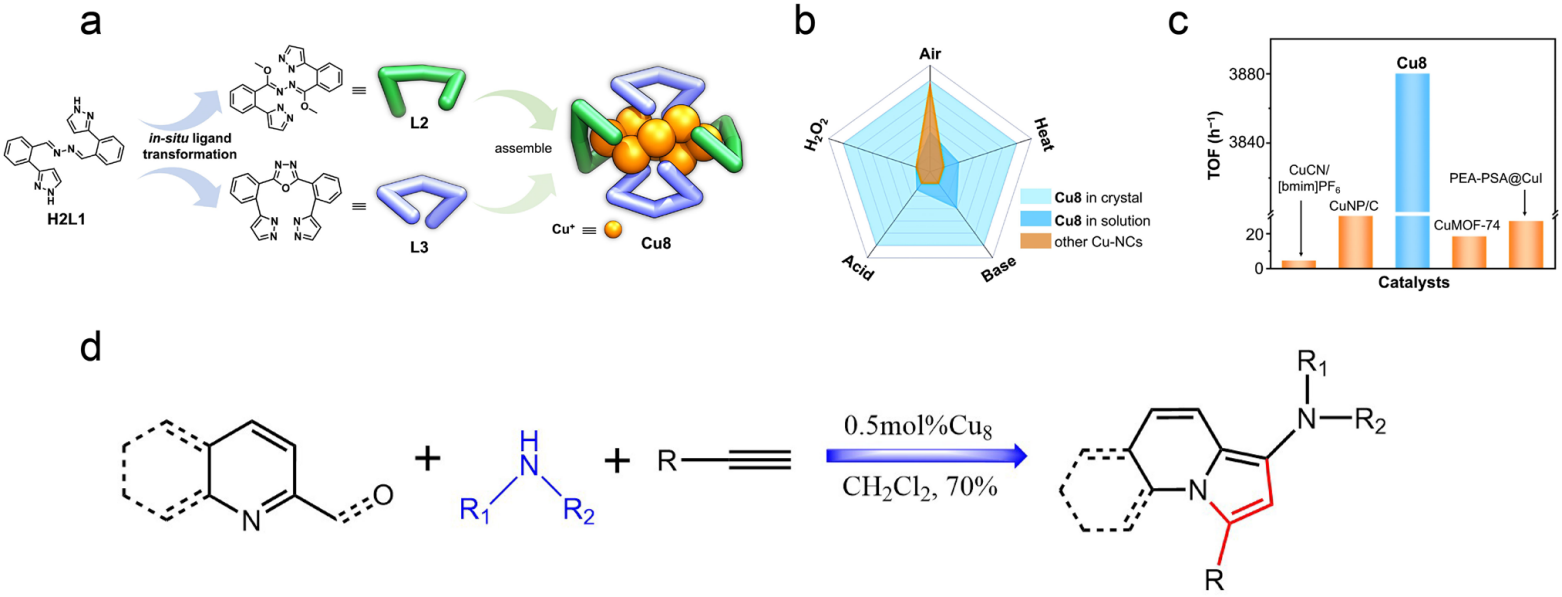
**a** Schematic illustration for preparing pyrazolate-protected Cu_8_ nanocluster. **b** Radar chart of various factors for the guidance and assessment of chemical stability of Cu_8_ and other Cu-NCs. **c** TOF comparison for the indolizine synthesis based on Cu_8_ and other catalysts. **d** Indolizines synthesis catalyzed by Cu_8_ nanoclusters. Reproduced with permission from Ref. [62], Copyright 2023 Wiley VC

It is worth noting that most ligand protected Cu nanoclusters feature a core–shell framework hence the active sites can be blocked by metal–ligand shell or surface passivation units. Therefore, a strategy that can largely expose the metal active sites is long pursued for catalytic coupling reaction. In 2023, Xu et al. reported a novel approach to construct array-based ([Cu_8_(Tf-dpf)_4_(NO_3_)_2_](NO_3_)_2_ clusters [61]. Compared with the ([Cu_8_(RS)_6_(PPh_3_)_4_(MeCN)_4_H]^+^ with the cubic core, the above array based Cu_8_ cluster displays largely uncoordinated metal sites, hence exhibited significantly enhanced catalytic activity in the “aldehyde-acetylene-amine” A^3^-coupling reaction for synthesizing propargylamines. It shows the great potential to fabricate array-based Cu nanoclusters for A^3^ coupling reaction and beyond [61].

#### Synthesis for Indolizines

To realize the practical catalytic application of Cu nanoclusters, the long-term stability remains a long-standing challenge. In the regime of coinage metal nanocluster, compared with Au, Ag, and Au/Ag alloy clusters, the study of Cu nanoclusters lags far behind, mainly due to the high reactivity of Cu and low standard reduction potential (*E*^*0*^_Cu2+/Cu+_  = 0.34 V, *E*^*0*^_Cu+/Cu_ = 0.52 V) [22]. The surface organic ligand such as thiolate molecules, alkynes, halides, hydride can improve the stability due to the formation of strong chemical bonds, but the long-term stability for some specific organic reactions are still unsatisfactory. These ligands are considered as soft base, and Cu^2+^ or Cu^+^ ions belong to soft acid; therefore, the ligand–metal interaction fits the Pearson’s hard/soft acid/base theory [167].

In a recent study conducted by the Li and Ni team, pyrazolate (denote as Pz) ligands were employed to synthesize Cu_8_ nanoclusters [62]. Pz is a soft base, and thanks to the high p*K*_a_ value of 19.8 for deprotonated pyrazole [168], Pz protected Cu clusters are expected to exhibit exceptional alkali-resistant capability. As determined from SCXRD (Fig. 15a), the flexible bis-pyrazole ligands (H2L1) can transform into two novel deprotonated bis-pyrazole ligands (L2 and L3), and all these L2 and L3 ligands can assemble with Cu^+^ to yield Cu_8_ nanoclusters. The crystal of Cu_8_ clusters can keep intact in various organic solvents, highly concentrated acid, saturated alkali, oxidant, and boiling water, and still are fine for SCXRD test. More impressively, the introduction of organic acid or base (100 eq. HOAc, or 400 eq. dibutylamine) cannot impose structural decomposition, neither, confirming the remarkable stability never documented for Cu nanoclusters (Fig. 15b). Subsequently, the synthesis for indolizines from 2-pyridinecarboxaldehyde derivatives, terminal alkynes and secondary amines was tested using Cu_8_ nanocluster as a catalyst. Interestingly, the reaction cannot proceed without Cu_8_ cluster as the catalyst (Fig. 15c). The optimized condition is 0.005% mol of substrates. Other Cu nanoclusters such as Cu_10_ or Cu_18_ had a much lower yield, as they are not stable in basic conditions (Fig. 15d). Consequently, the turnover frequency (TOF) of Cu_8_ nanoclusters can reach 3880 h^−1^, about 3 orders of magnitude larger than the reported catalysts toward the synthesis of indolizines [62]. This study provides a new strategy to synthesize pyrazolate-protected Cu nanoclusters with ultrahigh chemical stability for practical applications.

#### Reduction of Ferricyanide to Ferrocyanide

Ferricyanide ([Fe(CN)_6_]^3−^) is an environmentally unfriendly material that readily reacts with acids to produce toxic hydrogen cyanide gas. The catalytic reduction of [Fe(CN)_6_]^3−^ to ferrocyanide ([Fe(CN)_6_]^4−^) is one effective strategy to limit the toxicity of [Fe(CN)_6_]^3−^ [169].

In a recent study, the Luo and Sun team reported the Cu_18_H(PET)_14_(PPh_3_)_6_(isothiocyanate)_3_ (Cu_18_H in short) nanocluster [63], which exhibits excellent catalytic performance in the reduction of ferricyanide. Cu_18_H comprises a pseudo D3-symmetrical triple-stranded helical Cu_15_ kernel, and can be structurally described as layer-by-layer combination of multiple chiral Cu nanoclusters linked through copper-thiolate bonds. Remarkably, the aggregate state of the Cu_18_H nanoclusters can catalyze electron transfer reactions efficiently. To assess its catalytic capability, ultraviolet/visible absorption spectroscopy was used to monitor the reduction process. When Cu_18_H and NaBH_4_ were added simultaneously, [Fe(CN)_6_]^3−^ can be completely converted into [Fe(CN)_6_]^4−^ very rapidly, and the solution turned into colorless within 14 s. In a control test, the reduction reaction of [Fe(CN)_6_]^3−^ without Cu_18_H required 16 min. It indicates that Cu_18_H has good catalytic reactivity for the reduction of [Fe(CN)_6_]^3−^ [63]. This study makes an interesting model for investigating and elucidating the aggregated state of copper nanoclusters.

#### Hydrogenation of Cyclohexanone

The chemical hydrogenation of cyclohexanone is among the most important and prevalent transformations in industrial organic synthesis, thanks to its effectiveness and economic viability [170, 171]. A previous report suggested that copper hydride nanoclusters are very promising for catalyzing the hydrogenation of carbonyl compounds, despite that there are challenges to control the product selectivity [172].

In a recent work done by Sun et al., a cluster of [Cu_66_Cl_8_(PPh_3_)_8_(SC_2_H_5_)_32_H_24_](SbF_6_)_2_ (Cu_66_ in short) was synthesized and its catalytic performance toward the hydrogenation of cyclohexanone was examined [64]. Cu_66_ contains an orderly of 16 Cu_4_ squares, and the ligands coordinate to the surface of the cluster in a regiospecific manner, displaying square pattern as well. To enhance the monodispersity and stability of the cluster catalyst, the Cu_66_ nanoclusters were immobilized on carbon black (XC-72) to afford the Cu_66_/XC-72 catalyst, and it demonstrated an unprecedented performance toward the hydrogenation of cyclohexanone. The selectivity of cyclohexanol is nearly 100%. Under specific conditions, complete conversion of cyclohexanone to the desired product was achieved within 40 h. The authors also tested all the other Cu catalysts, including (Cu(CF_3_CO_2_)_2_, CuCl, Cu_54_Cl_12_(NO_3_)_12_(SC_4_H_9_)_20_S (Cu_54_), Cu_50_(PhCOO)_10_(4-F-PhS)_20_(PPh_3_)H_2_ (Cu_50_), SeCu_20_(PhSe)_12_(PPh_3_)_2_(C_6_H_5_COO)_6_ (Cu_20_), and [Cu_25_(SPhCl_2_)_18_H_10_]^3−^ (Cu_25_), but they showed significantly lower yields for the corresponding product. Moreover, the Cu_66_/XC-72 catalyst exhibited no significant decay in cyclohexanol product selectivity, highlighting the robustness of the Cu_66_/XC-72 catalyst [64]. This study showcases Cu nanoclusters have high stability and exceptional catalytic activity in the hydrogenation of carbonyl compounds, envisioning a bright future in both fundamental research and practical applications.

### Cu NCs for Other Reactions

Atomically precise Cu nanoclusters can also find versatile catalytic applications toward other chemical reactions. One typical reaction is the catalytic hydrogenation of nitroarene. It is worth noting that the catalytic hydrogenation of toxic nitroaromatics into non-toxic amino molecules is a quite efficient and economical method, as amino molecules are valuable intermediates for pharmaceuticals and dyes [169, 173, 174]. Coinage metal nanoclusters have been emerging as a new type of important hydrogenation catalysts, and the catalytic hydrogenation of 4-nitrophenol into 4-aminophenol in the presence of BH_4_^−^ reducing agent has been employed as a model reaction in several previous studies [32, 92, 95, 175].

**Fig. 16:**
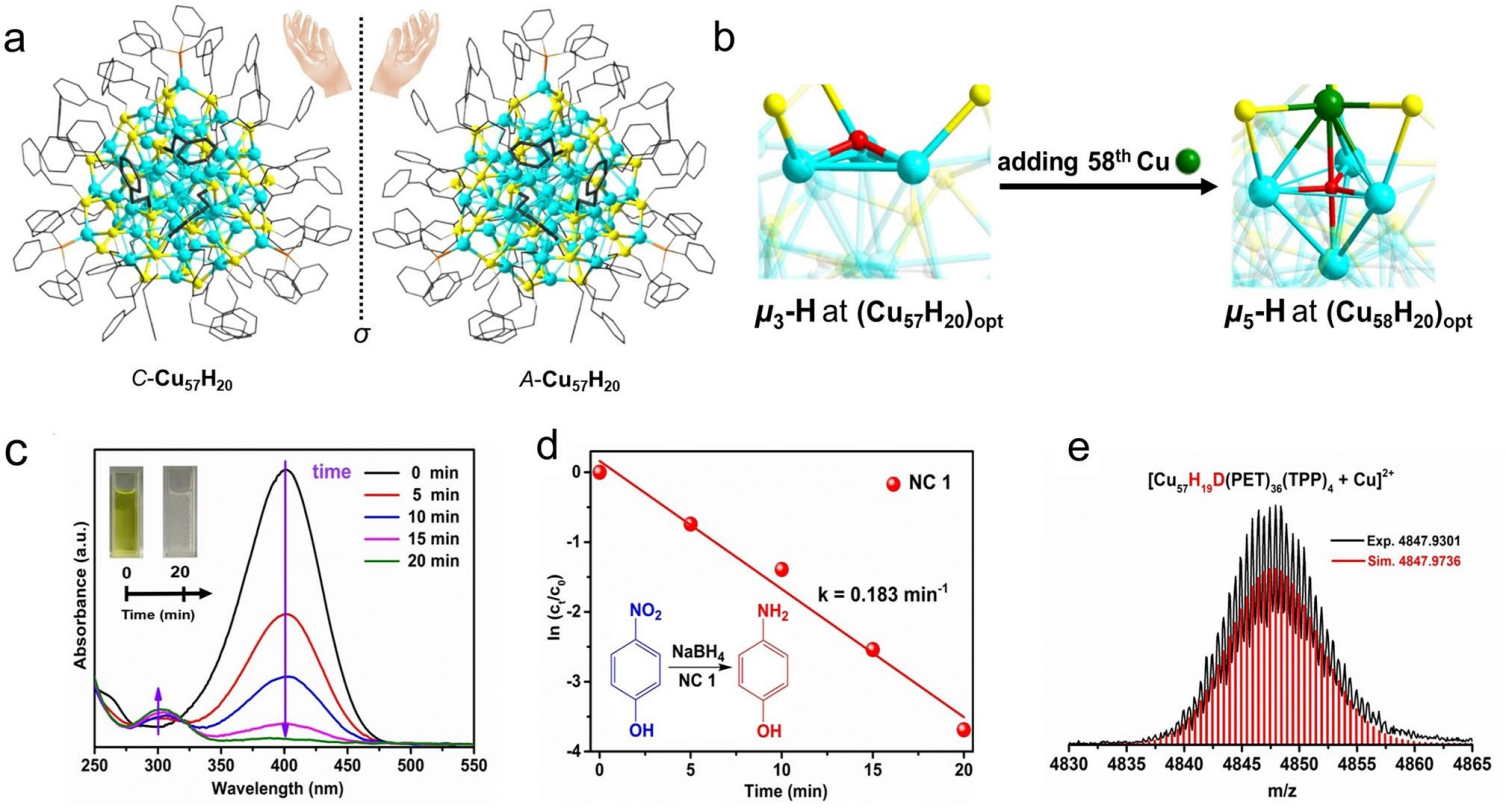
**a** Total structures of the C–Cu_57_H_20_ and A-Cu_57_H_20_ isomers. **b** Transformation of a *μ*_3_-H in (Cu_58_H_20_)_opt_ into a *μ*_5_-H in (Cu_58_H_20_)opt. **c** Absorption spectra versus time. **d** Plot of ln(C_t_/C_0_) versus time for 4-nitrophenol reduction. **e** ESI–MS spectrum after catalytic reaction with NaBD_4_. Color labels: Cu, turquoise; C, gray; P, orange; S, yellow; red, H; yellow, S. Reproduced with permission from Ref. [56], Copyright 2023 Wiley–VCH

In 2023, Luo et al. reported an intrinsically chiral Cu hydride-rich nanocluster of [Cu_57_H_20_(PET)_36_(TPP)_4_]^+^ (Cu_57_H_20_) and its catalytic activity toward reduction of 4-nitrophenol [56]. Single crystal X-ray diffraction measurement shows that the unit cell of the Cu_57_H_20_ cluster contains a pair of enantiomers, C-Cu_57_H_20_ and A-Cu_57_H_20_ (C, clockwise; A, anti-clockwise) (Fig. 16a). The hydride positions were further validated by DFT optimization of the model cluster of (Cu_57_H_20_)_opt_. Interestingly, the DFT analysis also suggests that the interfacial μ_3_-H in (Cu_57_H_20_)_opt_ can be transformed into an interstitial μ5-H species in the perfect cluster of (Cu_57_H_20_)_opt_ as long as the vacant position is recovered by the 58th Cu atom (Fig. 16b). The Cu_57_H_20_ cluster was then employed as the catalyst to catalyze the reduction of 4-nitrophenol. The reaction process was monitored by UV–vis absorption spectra (Fig. 16c), where strong absorbance peak at 400 nm from 4-nitrophenol decreased rapidly in 20 min and the absorbance peak at 300 nm from 4-aminophenol increased. The bright yellow solution also turned colorless. Using the absorbance change at 400 nm as metric, the reduction adopts a pseudo-first-order reaction kinetics, and the reaction rate was calculated as 18 min^−1^ (Fig. 16d). A control experiment in the absence of Cu_57_H_20_ was also conducted, and no reduction occurs on 4-nitrophenol when adding BH_4_^−^, probably due to the high activation barrier between the repelling 4-nitrophenol anion and BH_4_^−^ [176]. Finally, the authors performed a 4-nitrophenol reduction test by using BD_4_^−^ to check the accessibility of the hydride in Cu_57_H_20_. After the catalytic test, the cluster was examined by the ESI–MS measurement (Fig. 16e). It shows well defined peak feature of [Cu_57_H_19_D(PET)_36_(TPP)_4_ + Cu]^2+^, indicating one H^−^ is indeed replaced by one D^−^ of BD_4_^−^, that means, the exposed interfacial μ_3_-H might be involved in the catalytic reaction [56]. By using defective Cu_57_H_20_ cluster as model catalyst, this work offers atom-precise insights into the vacant defect role in catalysis.

In addition, a very small change in composition can make a big difference in structure and catalytic performance for some specific catalytic reactions. In a recent report, the Wang and Jiang team disclosed the dramatic difference between Cu_20_H_8_ and Cu_20_H_9_ clusters in catalysis [65]. Specifically, two copper hydride clusters, [Cu_20_H_9_(Tf-dpf)_10_]·BF_4_ (Cu_20_H_9_) and [Cu_20_H_8_(Tf-dpf)_10_]·(BF_4_)_2_ (Cu_20_H_8_) (Tf-dpf = N, N′-di(5-trifluoromethyl-2-pyridyl)formamidinate) were fabricated, and both have twenty Cu atoms and ten amidinate ligands but with one hydride difference in the ligand shell. Such differences led to drastically different geometric and electronic structures, resulting in different catalytic properties. Cu_20_H_8_ showed 25 times higher catalytic activity than Cu_20_H_9_ in the conjugate reduction of cinnamaldehyde, mainly due to the easier dissociation process of a Tf-dpf^−^ ligand in Cu_20_H_8_ [65]. This work highlights the sensitivity of structure and composition toward catalytic reactions by using atomically precise copper hydride nanoclusters as catalysts.

Recently, the Shen group found that the open metal site plays a critical role in the catalytic reduction of 4-nitrophenol by using two Cu_41_ nanoclusters as model catalysts [46]. Specifically, two isostructural Cu nanoclusters of [Cu_41_Cl_2_(2-FC_6_H_4_S)_12_(CF_3_COO)_6_(PPh_3_)_6_H_19_]^2−^ (1H) and [Cu_41_(2,5-di-Methyl-C_6_H_3_S)_12_(BO_3_)_3_Cl_3_(PPh_3_)_6_H_19_] (2H) were prepared and fully characterized, and the main structural difference of them lies in the absence or presence of two additional chlorides on the surface, which in turn heavily governs the exposure of metal sites. The 2H cluster with more open active sites exhibited ~ sixfold increase of rate constant in the catalytic reduction of 4-nitrophenol than 1H cluster [46]. This work manifests that atomically precise Cu nanoclusters can serve as catalyst models to directly visualize the active sites that drive the chemical transformation. Meanwhile, the same group also discovered that the small distinctions in two carboxylate-protected Cu_20_ clusters can cause distinct catalytic performance toward 4-nitrophenol reduction [66]. Two clusters Se@Cu_20_(PhSe)_12_(PPh_3_)_2_(C_6_H_5_COO)_6_ (Cu_20_-1) and Se@Cu_20_(PhSe)_12_(PPh_3_)_2_(CF_3_COO)_6_ (Cu_20_-2) share with the identical metal skeleton and similar ligand distributions, and the main difference is the carboxylate ligand: C_6_H_5_COO^−^ for Cu_20_-1 while CF_3_COO^−^ for Cu_20_-2. Consequently, Cu_20_-1 has a catalytic activity enhancement of 16-fold than Cu_20_-2. Such catalytic performance distinction is attributed to the carboxylate effect, that is, the functional group (-C_6_H_5_CO_2_ or -CF_3_CO_2_) altered the electronic structure of the Cu nanocluster [66].

## Challenges and Perspectives

The last decade, especially the recent five years, have witnessed great success in atomically precise Cu nanoclusters, including synthesis, structural analysis, property exploration, and various catalytic applications. Despite the significant progress, there are still some important obstacles for nourishing the atomically precise Cu nanocluster field, especially in both synthetic regime and catalytic field. Some possible challenges and future perspectives are discussed below, aiming to motivate or advance this fast-growing field:

1. High-efficiency synthetic method to obtain atomically precise Cu nanoclusters is still highly desired. Compared with the fruitful achievements in Au and Ag nanoclusters, the synthesis and total structure determination of Cu nanoclusters have been lagging behind, probably due to the variability of Cu valence states. It is known that the subtle reaction conditions may affect the output of the final product, e.g., the solvent, the reaction temperature, the ligand (discuss below), the reducing agent, the pH value in the reaction system, the stoichiometric ratio of all the reactants, etc. For example, by using CH_2_Cl_2_ as the solvent, the Cl atoms can coordinate with Ag and become the capping ligand in alkynyl-protected Ag_112_ nanoclusters [177], and it might also be applicable for preparing Cu nanoclusters. More importantly, the current synthetic methods of atomically precise Cu nanoclusters are heavily dependent on the “the-trial-and-error” efforts, which are quite tedious, expensive, and low efficient. High throughput synthesis with the aid of artificial intelligence or machine learning technique represents an embryonic avenue yet still have a long way to go [178, 179].
2. The role of ligand should be carefully considered. The choosing of ligand molecule not only affects the synthesis, but also plays a critical role in the catalytic process. Thiolate, alkynyl molecule, hydride, halogen, phosphine, N-heterocyclic carbene, inorganic anions (e.g., BF_4_^-^, CH_3_COO^-^) are most widely employed ligand molecules for stabilizing Cu nanoclusters, but they possess different binding strength to the Cu core. It is commonly believed, the surface ligand can block some active sites for catalysis [161], as they can inhibit the reactants for accessing the metal sites. However, more and more cases have shown that, the ligand can facilitate the catalytic process, e.g., accelerate the transfer of the reactants or intermediates via weak interactions. Weak surface ligand is more easily stripped off or exchanged by the reactant to create catalytic sites [10]. Moreover, the type of ligand molecule affects the catalytic activity drastically, e.g., Tsukuda group reported that alkynyl-protected Au_25_ clusters exhibited markedly higher HER activity than thiolate-protected Au_25_ molecules [180], while our group discovered that, the Faradaic efficiency of CO for alkynyl-protected Ag_32_ clusters is much higher than the thiolate and phosphine ligand co-protected Ag_32_ clusters in eCO_2_RR [32]. Such catalytic property difference is attributed to the electronic structure change owing to the electronic perturbation of the π-conjugated units [72]. Even with the same type of molecule in ligand, the steric hindrance and electronic structure can affect the catalytic properties, e.g., using bulky ligand may create some low coordinated or undercoordinated metal sites for catalysis [159, 181]. The Xie group found that, in the oxygen evolution reaction catalyzed by three thiolates protected Au_25_ nanoclusters, *p*-mercaptobenzoic acid stabilized Au_25_ nanoclusters exhibited markedly superior catalytic performance, simply because the ligand’s stronger electron-withdrawing ability can create more partial positive charges on Au(I) as active site for facilitating feasible adsorption of OH^-^ in alkaline media [182]. Yoo et al. found that the locally induced hydrophobicity by bulky alkyl functionality near the surface of the Ag_25_(SR)_18_ cluster dramatically enhanced the eCO_2_RR activity, where the hydrophobic Ag_25_ cluster exhibited remarkable selectivity for CO (FE_CO_ > 90%) and achieved a high current density of up to -240 mA cm^-2^ with excellent durability lasting for over 120 h [183]. Nevertheless, the ligand role of atomically precise Cu nanoclusters during the synthetic process and catalytic duration remains largely to be explored.
3. The metal core tailoring. The metal core configuration and atomic spatial arrangement can be critical for exposing available catalytic active sites for Cu nanoclusters. So far, there are core-shell, rod-like, array-like core structure and other core configurations for Cu nanoclusters, but a trade-off between activity, selectivity and stability must be taken into account for specific catalytic reactions [69]. More profoundly, more dedicated core structure engineering might be necessary for Cu nanoclusters. Vacancy engineering has demonstrated great potential, which is expected to catch more future research attention [58]. Another important direction is alloying other metals to form atomically precise Cu–alloy nanoclusters. For catalytic reactions, the size, composition, configuration, electronic structure are the main factors that influence the catalytic performance, and introducing another metal to form Cu–alloy can modulate all the above factors. Cu-based bimetallic nanoclusters have demonstrated unusual properties and extraordinary catalytic capabilities [90, 184], yet controlling the number and the exact position of a foreign metal atom remains challenging [163, 185]. Particularly, single-metal-atom doping have been gaining tremendous efforts in catalysis study but increasing the production yield for generating single-atom doping during the synthetic process, understanding the geometric and electronic structure change in the on-working catalytic process is essential for future high-performance catalyst design [186–189].
4. *In situ* reaction mechanism study. Understanding the reaction mechanism in on-working status is quite valuable but still challenging. The *in situ* and *operando* spectroscopic and synchronous techniques such as surface enhanced Raman scattering, infrared spectroscopy, high-energy X-ray diffraction (HE-XRD), extended X-ray absorption fine structure (EXAFS), X-ray absorption near edge structure (XANES), and small-angle neutron scattering (SANS) can provide the possibility of acquiring the structure of the catalytic intermediates, which can enhance the evidence for proposed reaction mechanism [190–193]. For instance, the *in situ* infrared spectroscopy was employed to capture the reaction intermediates during the electrocatalytic nitrate reduction catalyzed by Ag_30_Pd_4_ nanoclusters; hence, the tandem catalytic mechanism was successfully revealed by our group [93]. Nevertheless, the consecutive picture of the whole reaction process including adsorption, transfer, desorption of the reaction intermediates on Cu nanoclusters are quite limited in current studies, and more advanced *in situ*/*operando* study and the necessary instrumental modification for some specific *in situ/operando* investigations are expected.
5. The more precise theoretical simulations. Theoretical simulations can calculate the energy barrier of each step, acquire the optimal configuration between the cluster and the reactant, the intermediate, or the product, hence plays a critical role in speculating the active sites and deciding the rate-determining step [104]. However, the accuracy of these calculations depends on the authenticity and the extent of the structure proximity between the real crystal structure and the theoretical model. The fully ligand capped Cu nanoclusters are too complicated to build a real model, so the “ligand simplification” strategy is widely used. For instance, Au_25_(SCH_3_)_18_ has been used to replace Au_25_(SC_2_H_4_Ph)_18_, Au_25_(SC_6_H_13_)_18_ and Au_25_(SC_12_H_25_)_18_ in several cases for building theoretical models [194–196]. Authentic models can help to understand the true reaction process and gain more realistic reaction mechanism but given the complexity of the cluster structures and the reaction conditions, more precise and realistic theoretical simulations still have a long way to go.
